# Tuning Core Flexibility and Curvature in Azine-Linked Covalent Organic Frameworks for Attomolar-Level Impedimetric Sensing of Glucose

**DOI:** 10.1021/jacs.5c10578

**Published:** 2025-10-08

**Authors:** Nada Elmerhi, Sara Awni Alkhatib, Sushil Kumar, José Ignacio Martínez, Nabila Yasmeen, Najat Maher Aldaqqa, Blaž Belec, Anna-Maria Pappa, Dinesh Shetty

**Affiliations:** † Department of Chemistry, 105955 Khalifa University of Science and Technology, Abu Dhabi 127788, United Arab Emirates; ‡ Department of Biomedical Engineering and Biotechnology, Khalifa University of Science and Technology, Abu Dhabi 127788, United Arab Emirates; § Instituto de Ciencia de Materiales de Madrid (ICMM-CSIC). C/Sor Juana Inés de la Cruz 3, Madrid 28049, Spain; ∥ Materials Research Laboratory, University of Nova Gorica, Vipavska 11c, Ajdovscina 5270, Slovenia; ⊥ Center for Catalysis and Separations (CeCaS), Khalifa University of Science and Technology, Abu Dhabi 127788, United Arab Emirates; # Biotechnology Center, Khalifa University of Science and Technology, Abu Dhabi 127788, United Arab Emirates

## Abstract

Glucose serves as a key biomarker of metabolic health, requiring continuous and reliable monitoring. Nonenzymatic impedimetric sensors have emerged as a promising alternative to enzymatic glucose sensors, which dominate current clinical practice. However, their sensitivity is limited by weak molecular recognition and inefficient charge transport at the sensing interface. In this study, we tuned the core flexibility and curvature of azine-linked covalent organic frameworks (COFs) by substituting the central triazine ring in the C_3_ symmetric building unit with a nitrogen atom. This modification maximized glucose–framework interactions, thereby enhancing signal transduction and detection sensitivity. The results confirmed the sensitivity and selectivity of the COFs toward glucose, with a linear response over a wide concentration range [1 aM–5 mM], surpassing previously reported glucose sensors. The sensors achieved a subattomolar limit of detection (LOD), with excellent stability and reproducibility compared to traditional enzymatic sensors, offering a simpler and more practical alternative for integration into wearable and implantable devices. Experimental findings are supported by molecular simulations, which reveals the mechanism of sensing and charge transfer dynamics. This work establishes a versatile design strategy for enzyme-free designed-to-purpose transducers, opening new pathways for ultrasensitive and selective molecular detection.

## Graphical abstract

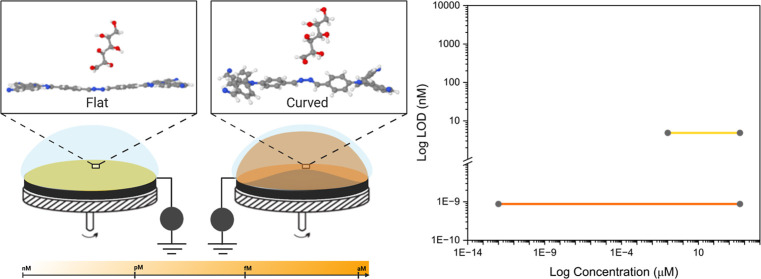

## 1 Introduction

Glucose is a crucial biomarker of metabolic health, closely associated with various physiological and pathological conditions including diabetes mellitus, cardiovascular disease, chronic kidney failure, and neuropathy.^1–4^ Commercial glucose biosensors rely on immobilizing glucose oxidase (GOx) on a carbon-based electrode to provide a selective recognition layer that specifically catalyzes glucose oxidation and converts its biochemical concentration into a measurable electrical signal via the electrode’s transducer.^5–7^ Electrochemical biosensors are characterized by their rapid response times, high sensitivity and selectivity toward specific analytes, while also being cost-effective, portable, and user-friendly.^8–10^ However, they suffer from complex enzyme immobilization procedures, enzyme leaching, limited accuracy, short-term stability, and high production costs.^11,12^ These aspects need to be considered when it comes to their practical implementation. In recent years, nonenzymatic electrochemical sensors have attracted considerable attention to detect analytes, notably glucose, with great progress.^13–15^ A broad spectrum of materials has been explored for this purpose, including precious metals (e.g., Pt, Ag, Au), nonprecious transition metals, metal oxides, composites, and other functional materials.^11,13,16–19^ However, most of the explored electrode materials suffer from narrow detection ranges, inadequate sensitivity, and limited selectivity in the presence of reactive biomolecules.^20^

Covalent organic frameworks (COFs), which are crystalline and porous polymers, have emerged as promising electrode materials for electrochemical sensing owing to their structural/functional tunability, permanent periodic porosity, and chemical stability.^21,22^ Still, reports on electrochemical glucose sensing using COFs remain limited, with all relying on enzyme- or metal-incorporated frameworks for the direct electro-oxidation of glucose at the electrode surface.^21,23–26^ While this sensing strategy offers simplicity and rapid response, it suffers from electrode fouling due to oxidation byproducts, interference from reactive species in complex biological media, and limited sensitivity, especially at trace levels.^20^ Impedimetric sensing using electrochemical impedance spectroscopy (EIS), on the other hand, offers exceptional sensitivity to changes at the electrode–electrolyte interface induced by molecular diffusion, adsorption, and charge transfer.^27–30^ However, the successful design of COFs for ultrasensitive and selective impeditive sensing of glucose requires a thorough understanding of the interplay between the availability of accessible active sites for effective interactions with glucose molecules and charge transfer dynamics.^31^

Herein, we substituted the central triazine ring of the C_3_ symmetric building unit with a nitrogen atom to induce a change in the COF’s electronic properties. In addition, this substitution alters the dihedral angles between the core and the peripheral phenyl rings of the C_3_ unit, resulting in a lower degree of planarity and greater flexibility. This structural modification serves two purposes: First, the introduction of nitrogen enhances the electron-donating ability of the core, increasing the framework’s overall conductivity and facilitating more efficient charge transfer at the electrode interface, both of which are crucial for electrochemical impedance-based detection.^32–35^ Second, the induced core flexibility and resultant curvature in the framework structure reduce excessive π–π stacking and enhance the exposure of accessible active sites by facilitating the formation of adaptive binding environments for glucose molecules. Such adaptability maximizes glucose–framework interactions while minimizing nonspecific adsorption and byproducts accumulation, thereby mitigating electrode fouling and enhancing detection sensitivity at trace levels.^34–39^

Encouraged by the aforementioned concept, we have synthesized azine-linked COFs by a Schiff-base condensation reaction of C_3_ aldehydes with hydrazine and explored the translation of structural and chemical variation in the precursor to the overall electronic properties, with the consequent influence on the impedimetric sensing of glucose. Our molecular-level design creates a spatially resolved electronic environment that enhances selective glucose binding, enabling the detection and quantification of glucose at trace levels. The findings reveal a significant enhancement in glucose sensing performance, surpassing previously reported sensors, with a broad range of detection spanning from 1 aM to 5 mM, a limit of detection (LOD) of 0.878 aM, and a limit of quantification (LOQ) of 2.66 aM. This unprecedented subattomolar sensitivity enables the detection of ultratrace glucose fluctuations, which are critical for early-stage disease monitoring. The sensor exhibited excellent selectivity, with minimal interference from common physiological interferents, including sucrose, galactose, lactic acid, uric acid, and ascorbic acid. Notably, the sensing system demonstrated long-term operational stability and reproducibility exceeding those of enzymatic counterparts. In addition, molecular simulations revealed the underlying sensing mechanism and charge transfer dynamics. Our work advances the design of nonenzymatic electrochemical glucose sensors, harnessing the inherent flexibility and curvature of azine-linked COFs to achieve high sensitivity and specificity in glucose sensing. This work broadens the scope of COFs in electrochemical sensing, introducing design-to-purpose frameworks that offer robust and practical solutions for medical diagnostics.

## 2 Results and Discussion

### 2.1 Synthesis and Characterization of COFs

The azine-linked COFs, TtaHz and TFPAHz, were synthesized by a mechanochemical Schiff base condensation reaction between 4,4′,4″-(1,3,5-triazine-2,4,6-triyl)­tribenzaldehyde (Tta) or tris­(4-formylphenyl)­amine (TFPA) and hydrazine (Hz) in the presence of p-toluenesulfonic acid (PTSA) as a catalyst, followed by thermal treatment at 90 °C for 24 h (Figures 1a, S1 and S2). The sharpness in the powder X-ray diffraction (PXRD) patterns of the as-synthesized COFs indicates the formation of crystalline long-range ordered framework structures. The PXRD profile of TtaHz reveals the presence of three distinct diffraction peaks at 2θ values of 3.7°, 6.1°, and 9.5°, corresponding to reflections from (100), (110), and (120) planes, respectively (Figure 1b). In addition, a broad peak is detected from the reflection of (001) plane (centered at 2θ = 26.6°), which is an indication of π–π stacking of the COF layers.^40^ Meanwhile, the TFPAHz profile shows three diffraction peaks at 4.4°, 6.9°, and 11.7° (Figure 1c). Notably, the stacking peak associated with interlayer *d*-spacing in the PXRD profile of TtaHz is absent in TFPAHz, suggesting that the conformational distortion of the TFPA units weekens interlayer interactions. The experimental PXRD patterns of both COFs match well with the simulated patterns obtained from structural simulations coupled with Pawley refinements, confirming that the proposed structures adopt eclipsed AA stacking models (Figures 1b,c, S3 and S4). The interlayer distances were calculated to be 0.35 and 0.37 nm for TtaHz and TFPAHz, respectively (Figure 1b,c). This variation arises from the difference in structural planarity. The three phenyl rings connected to the central triazine core in TtaHz are coplanar with the core, exhibiting equal dihedral angles of 180°, reflecting a planar conformation that minimizes torsional strain. In contrast, the peripheral phenyl rings linked to the central nitrogen atom in TFPAHz deviate from planarity, with torsion angles of 23.55°, 25.07°, and 26.42° (Figure 1d,e). Therefore, the variation in planarity as a function of dihedral angles between the aldehyde precursor’s core and peripheral phenyl rings translates well into the structures of the resulting COFs (Figures S5 and S6).

**Figure 1:**
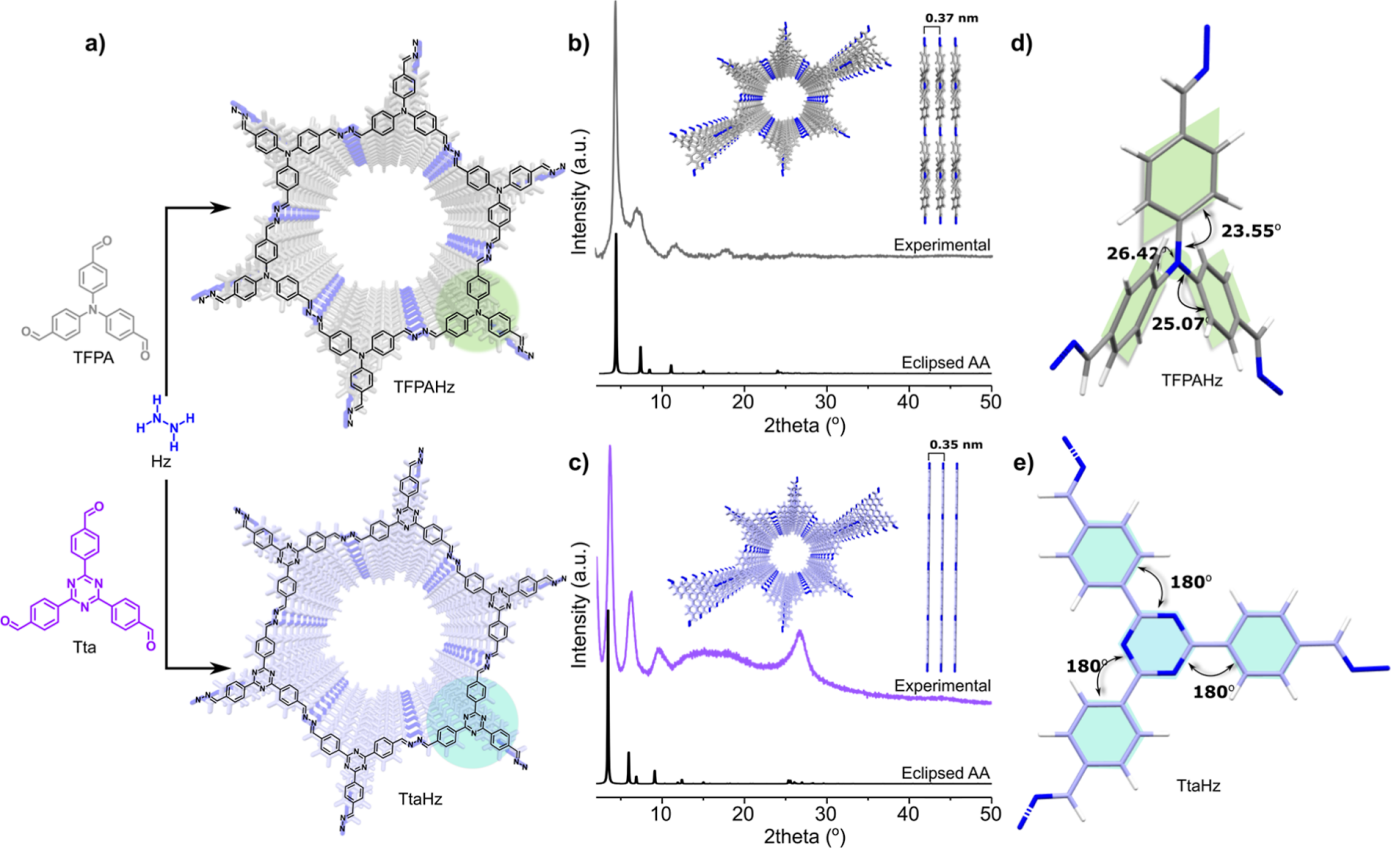
(a) Synthetic scheme of TFPAHz and TtaHz. Experimental PXRD pattern of (b) TFPAHz and (c) TtaHz compared to their simulated patterns for eclipsed AA stacking models. Dihedral angles between the core and the peripheral phenyl rings in (d) TFPAHz and (e) TtaHz, highlighted with double arrows to indicate the effect of core substitution on the framework’s planarity.

The Fourier transform infrared (FTIR) spectra show the appearance of the azine C=N stretching band (1619 cm^–1^), confirming the successful condensation and formation of the COFs (Figures 2a and S7 and S8). This is further supported by the significant attenuation in the intensities of the aldehyde C=O stretching bands (1677 cm^–1^–1693 cm^–1^) in the corresponding aldehyde precursors. The solid-state ^13^C Cross-Polarization/Magic Angle Spinning Nuclear Magnetic Resonance (^13^C CP/MAS NMR) spectroscopy confirms the structures of the COFs at the atomic level (Figure 2b). Both TtaHz and TFPAHz display the characteristic azine carbon (C=N) signal at ∼162 ppm, further attesting to the formation of the COFs. In addition, the signal at ∼169.4 ppm in TtaHz corresponds to the C=N in the triazine units, which is absent in TFPAHz.

**Figure 2:**
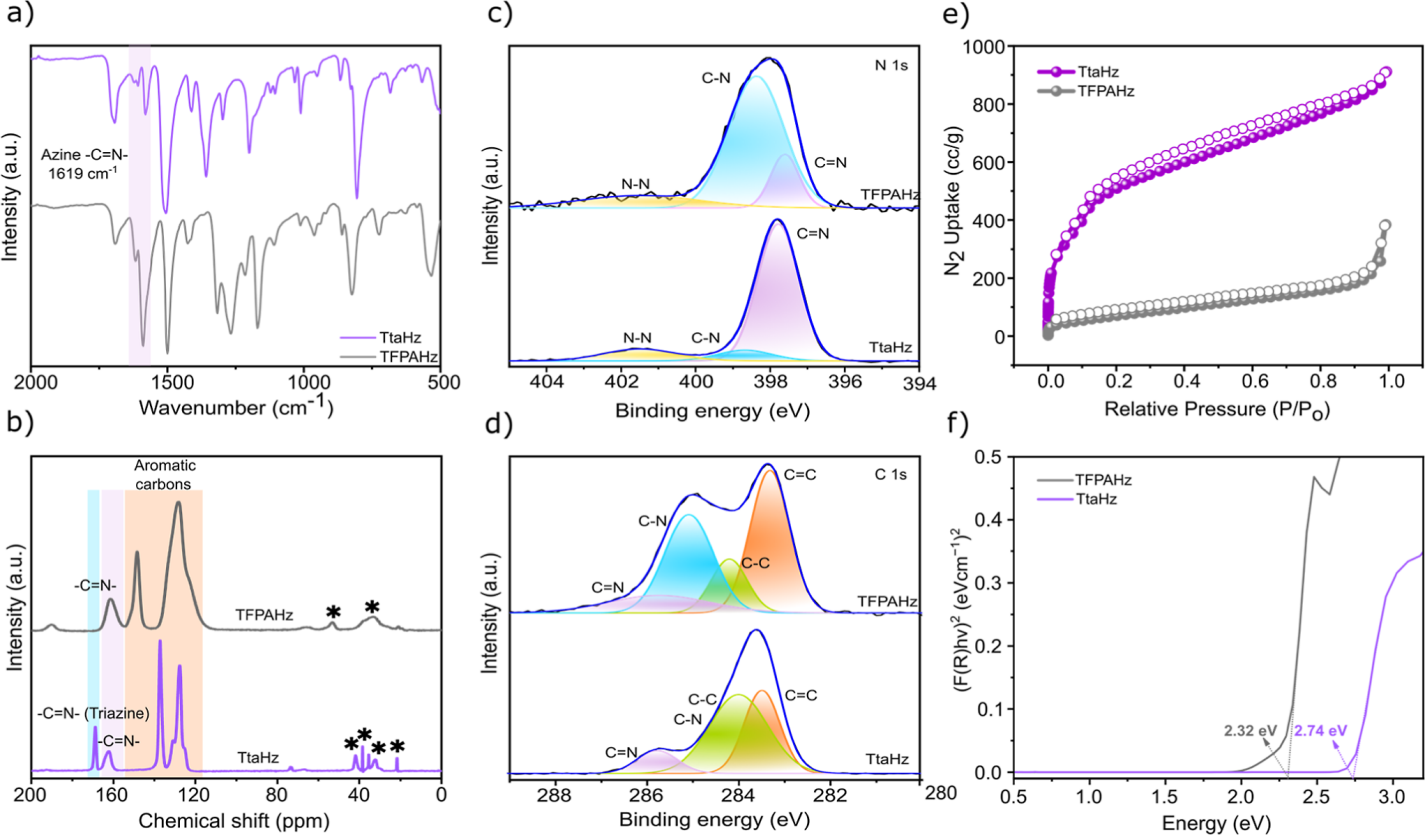
(a) FTIR of TtaHz and TFPAHz. (b) ^13^C CP/MAS NMR spectra of TtaHz and TFPAHz (* indicates spinning sidebands). (c) High-resolution deconvoluted (c) C 1s and (d) N 1s XPS spectra of TtaHz and TFPAHz. (e) Nitrogen gas adsorption–desorption isotherms of TtaHz and TFPAHz. (f) Band gap of TtaHz and TFPAHz.

The X-ray photoelectron spectroscopy (XPS) survey spectra of TtaHz and TFPAHz exhibit characteristic peaks at binding energies (BE) of 284.5, 398.6, and 532.6 eV, corresponding to C 1s, N 1s, and O 1s, respectively. The intensity of the N 1s peak is higher in TtaHz than TFPAHz, suggesting triazine groups’ additional presence (Figure S9). The high-resolution deconvoluted N 1s XPS spectra of TtaHz and TFPAHz reveal three peaks at 397.6, 398.4, and 401.5 eV originating from the azine C=N, C–N, and N–N, respectively (Figure 2c). The deconvoluted C 1s XPS spectra of both COFs revealed the presence of aromatic C=C, C–C, C–N, and C=N at BE values of 283.3, 284.2, 285.1, and 285.7 eV, respectively (Figure 2d). Scanning electron microscopy (SEM) images reveal that TtaHz forms irregular aggregates interspaced with elongated sheet-like structures, whereas TFPAHz exhibits thin, wrinkled sheets that assemble into a flower-like structure (Figure S10). Transmission electron microscopy (TEM) images support the SEM observations, confirming that TtaHz is composed of aggregates integrated within stacked lamellar layers, while the flower-like structure of TFPAHz arises from thin sheets with wrinkled and partially folded regions (Figure S9). Energy-dispersive X-ray spectroscopy (EDS) by SEM confirms the uniform carbon and nitrogen distribution throughout the framework structures (Figures S12 and S13). Notably, TtaHz exhibits a nitrogen content of 14.216 wt %, which is more than three times higher than that of TFPAHz (4.230 wt %), reflecting the difference in their composition (Table S1).

The N_2_ adsorption–desorption isotherms indicate that TtaHz and TFPAHz possess Brunauer–Emmett–Teller (BET) surface areas of 1987.3 m^2^ g^–1^ and 279.5 m^2^ g^–1^, respectively (Figure 2e). The significantly higher surface area observed for TtaHz than TFPAHz could be due to the differences in framework's planarity and stacking order. The planar, rigid triazine core in TtaHz facilitates tighter π–π stacking and denser pore packing, resulting in a highly porous framework.^41–43^ The nonlocal density functional theory (NLDFT) analyses reveal that TtaHz exhibits a narrow pore size distribution, centered at around 3.5 nm, whereas TFPAHz shows a broader and bimodal distribution with two average pore sizes centered around 1.4 and 2.8 nm (Figures S14 and S15). The experimentally determined pore sizes show excellent agreement with the theoretically calculated values. Both TtaHz and TFPAHz showed thermal stability up to 350 °C with less than 10% weight loss (Figure S16). The optical band gaps were calculated from the solid-state UV–Vis diffuse reflectance spectra using the Tauc plot method. The band gap for TtaHz and TFPAHz was calculated to be 2.74 and 2.32 eV, respectively (Figure 2f). The narrower bandgap of TFPAHz compared to TtaHz arises from the strong electron-donating character of the TFPA units, which enhances π-electron delocalization when coupled with the azine linkage, a mild electron acceptor. This donor–acceptor configuration reduces the HOMO–LUMO energy separation, facilitating greater intraframework charge delocalization. In contrast, TtaHz features triazine-containing units, which are known for their electron-deficient nature. Coupling these units with azine linkages results in a limited donor–acceptor contrast and interrupted conjugation that further suppresses electronic delocalization throughout the framework structure.^32–35^ The UV–vis absorption spectra further support these findings by revealing a pronounced red-shifted absorption onset in TFPAHz relative to TtaHz, consistent with its stronger donor–acceptor interactions and extended π-conjugation (Figure S17). Electronic modulation, coupled with the framework’s curvature, was deliberately introduced to enhance interfacial conductivity and molecular recognition, enabling ultrasensitive glucose detection and guiding the design of COF-based biosensing interfaces.

### 2.2 Preparation and Characterization of Sensing Electrodes

Homogeneous dispersions of the two COFs were prepared and uniformly deposited onto screen-printed carbon electrodes (SPCEs) using a spin-coating technique. Optimization of spin-coating parameters, including speed, duration, and stages, resulted in uniform and stable COF coatings on the surface of the electrodes (Figure 3a). The coatings adhered well to the SPCE surfaces without cracking or peeling off during the measurements. The SEM images confirm that both COFs retained their morphology after deposition on SPCEs, indicating that the coating process did not alter their structural integrity (Figure S18). Notably, the COF-coated electrodes showed excellent stability, with no observable electrochemical degradation after 72 h under ambient conditions (Figure S19). In addition, the coatings were stable after performing 10 cycles of electrochemical measurements (Figure S19). The long-term functional stability of the electrodes was also evaluated over a period of 7 days, during which only minimal variation was observed (Figure S20), confirming the reliable electrochemical response, and supporting their practical applicability for nonfaradaic glucose sensing.

**Figure 3:**
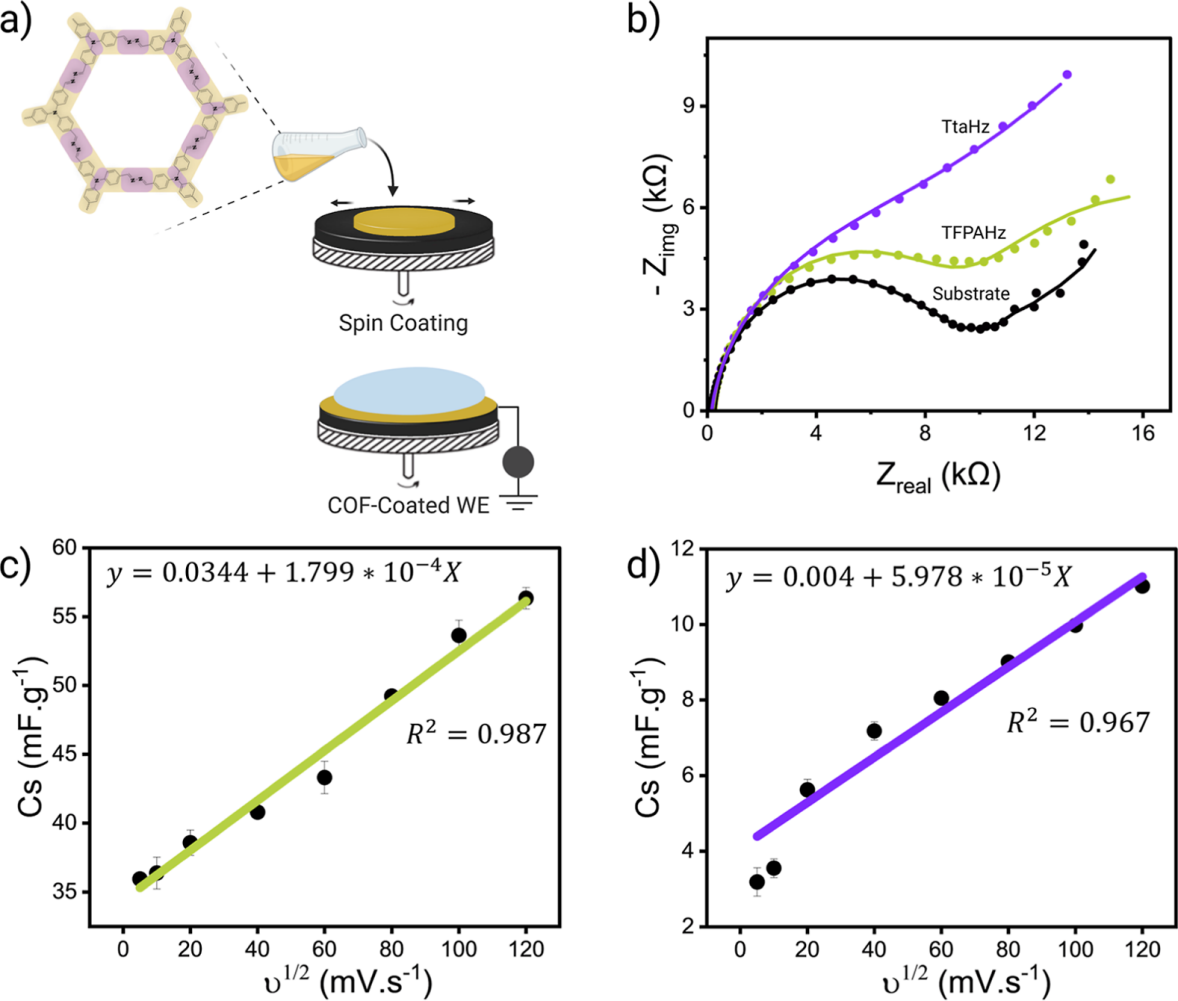
(a) Schematic representation for the preparation of the COF-coated electrode using spin coating technique. (b) EIS measurements for the bare electrode and the two COF-coated electrodes vs Ag/AgCl in 5 mM K_3_[Fe­(CN)_6_]. The plot shows the overlay of the experimental data and the simulated Nyquist plots fitted using the equivalent circuit. Calibration curve demonstrating the linear relationship between specific capacitance (Cs) and the square root of scan rate (υ^1/2^) for (c) TFPAHz-coated electrode and (d) TtaHz-coated electrode.

The electrochemical impedance spectroscopy (EIS) data revealed distinct differences in the charge transfer resistance (*R*_ct_) and other interfacial properties between TFPAHz-coated SPCE and TtaHz-coated SPCE. The trend observed in Figure 3b reveals the progressive influence of the different COFs on the interfacial properties of the electrode. The Nyquist semicircle increases upon coating the SPCE, reflecting a higher *R*_ct_ (Figure 3a,b), which could be due to the COF layer acting as a barrier that hinders electron transfer. TtaHz exhibited the largest semicircle (Figure 3b), reflecting the highest *R*_ct_ among the three configurations, likely due to the electron-deficient triazine core, which reduces charge carrier density and hinders interfacial electron transfer.

The Bode and phase plots further support these findings, with a clear increase in impedance magnitude (*Z*) and enhanced capacitive behavior upon coating the electrodes (Figure S21). These results suggest that the COFs not only impact *R*_ct_ but also enhance the overall capacitive properties of the SPCE electrode, likely due to their influence on interfacial charge distribution and electrochemical double-layer formation.

The capacitive properties were further investigated using CV measurements at different scan rates (5 to 120 mV/s) in Dulbecco’s phosphate buffer saline (DPBS) (Figure S22). The COF-coated electrodes exhibited uniform charge distribution and stable specific capacitance across all scan rates (Figure 3c,d). However, the overall capacitance values for TFPAHz were consistently higher than TtaHz, suggesting the enhanced charge storage capacity of TFPAHz. The *C*_s_ value for TFPAHz at 5 mV/s is around an order of magnitude higher than that of TtaHz (0.03549 and 0.00304 F, respectively), with a gap that keeps widening further with increasing scan rates (reaching 0.05606 and 0.01094 F, respectively), demonstrating that the TFPAHz-coated electrode provides a more efficient and stable interface for charge transfer. These observations could be attributed to the inherent structural and electronic differences between the two COFs. The strong electron-donating nature of TFPA in TFPAHz facilitates charge delocalization, reducing charge-transfer resistance and enhancing conductivity. In addition, the presence of TFPA introduces a curvature in the framework structure, which enhances interfacial contact with the electrolyte, optimizing charge accumulation at the interface of the double layer and leading to greater capacitive behavior. Overall, the electrochemical analyses reveal that TFPAHz offers superior electrochemical performance compared to TtaHz, as evidenced by the lower *R*_ct_ with extended Warburg tail and higher specific capacitance values. These findings reflect the potential of TFPAHz to be used for sensing applications.^23^

### 2.3 Glucose Sensing

EIS measurements were performed using SPCEs modified with the two COFs to assess their glucose sensing performance (Figures 4a,b and S24a). The impedance spectra display consistent and concentration-dependent changes upon glucose addition, most notably reflected by a progressive increase in the double-layer capacitance (C_dl_). In general, C_dl_ is more sensitive to changes in the electrochemical double layer formed at the electrode surface. As glucose molecules interact with the electrode, they alter the charge distribution at the interface, leading to a change in the capacitance, which could be quantitatively related to the glucose concentration.^44^ The TFPAHz-coated electrode exhibited a progressive, linear increase in C_dl_ across a wide concentration range [1 aM–5 mM], highlighting the outstanding sensitivity for glucose detection down to ultratrace levels (Figures 4c and S23). The theoretical C_dl_ increases from 0.67 to 3.98 mF/cm^2^ over the entire concentration range. The sensor achieved an LOD of 0.878 aM and LOQ of 2.66 aM (Table S2), surpassing previously reported glucose sensors (Table S4). While our current focus was on low-concentration detection to showcase the sensing capability of the material, we show in Figure S34 the material performance on physiologically relevant blood glucose concentrations [0.5–20] mM. The EIS measurements carried out in the higher glucose concentrations demonstrate a clear and reproducible change in C_dl_ values with increasing glucose concentration, which indicates that the relative sensitivity remains constant across the entire tested range.

**Figure 4:**
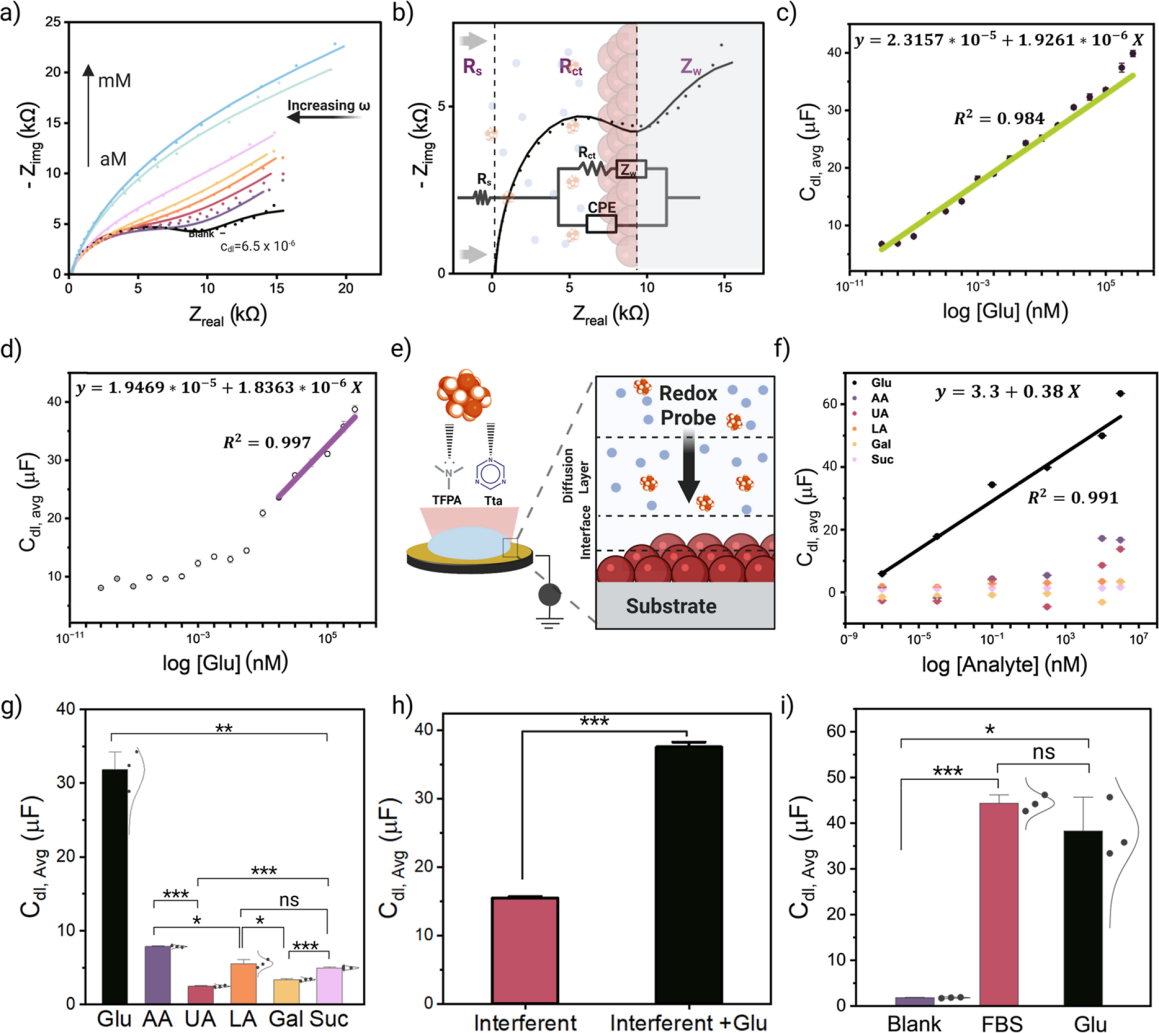
(a) Overlay of the experimental data of TFPAHz-coated electrode and the simulated Nyquist plots fitted using an equivalent circuit in response to increasing glucose concentration vs Ag/AgCl in 5 mM K_3_[Fe­(CN)_6_]. (b) Overlay of the experimental data of the TFPAHz-coated electrode in 5 mM K_3_[Fe­(CN)_6_] and the simulated Nyquist plot fitted using an equivalent circuit representing the determination of the solution resistance (*R*_s_), resistance to the charge transfer (*R*_ct_), and Warburg resistance (*Z*_w_) with schematic of the equivalent circuit used for fitting the Nyquist plots obtained from EIS measurements. Calibration curves showing the double-layer capacitance (C_dl_) values at different glucose concentrations with the fitted equation for (c) TFPAHz-coated electrode, and (d) TtaHz-coated electrode. (e) Schematic representation of the COF-coated electrodes during electrochemical measurement with corresponding structural interaction of both COFs with the glucose molecule and the interaction happening at the surface of the COF-coated electrode during glucose sensing measurement. (f) Calibration curves representing the average C_dl_ value (*N* = 3) for five different analytes against the analyte concentration. Parameters of fitted equations available in Table S2. (g) Selectivity of TFPAHz-coated electrode to glucose, the bar graph indicates the average C_dl_ for different analytes at 100 nM in 5 mM in K_3_[Fe­(CN)_6_], with calculated *p*-values. (h) Interferent effects (AA, UA, LA, and Suc) on the efficiency of glucose detection, the average C_dl_ value of interferent solution compared to interferent mixed with glucose. The final concentration of each analyte in the interferent solution is 100 nM. (i) Selectivity to glucose in FBS solution containing (∼1 μM) glucose prepared in K_3_[Fe­(CN)_6_] compared to 1 μM glucose and blank K_3_[Fe­(CN)_6_] solutions, with statistical significance determined by *p*-values. Statistical significance between groups is indicated as follows: ns = not significant, *p* ≤ 0.05, **p ≤ 0.01, ***p ≤ 0.001.

To assess the practical relevance and competitive advantage of the developed azine-linked COFs for glucose detection, the linear detection range and the LOD were mapped for the most recent nonenzymatic electrochemical glucose sensors using a wide range of similar material platforms to serve as a benchmark for evaluating our COF-based sensor (reported between [2017–2025], Table S4). Our COF-based glucose sensor surpasses the recently reported nonenzymatic systems, as shown in the comparative Figure S33 with an ultralow LOD and a wide linear detection range extending from 1 aM to 20 mM. The majority of reported sensors cluster within a higher LOD regime [10^–9^–10^–6^] M with narrower dynamic glucose concentration ranges. While most reported sensors offer physiologically sufficient performance in the micro and nanomolar range for a wide linear range, these values remain several orders of magnitude lower than the performance of our system, as they are insensitive to trace-level detection in low-abundance biofluids necessary for wearable, noninvasive diagnostics. On the other hand, the TtaHz-coated electrode showed a linear C_dl_ response to glucose concentration in the range of 100 nM to 5 mM, with an LOD of 4.87 nM and LOQ of 14.75 nM (Figure 4d and Table S2), suggesting a stable sensing behavior within a physiologically relevant range. SEM and TEM analyses were performed after glucose sensing to assess the microstructural stability throughout the sensing process. No significant morphological changes were observed, confirming that both azine-linked COFs retain their microstructural integrity under the sensing conditions (Figures S25 and S26).

Notably, the change in charge transfer resistance (*R*_ct_) for both COFs did not show a strong linear response to the same concentration ranges. Unlike the clear trends observed in EIS measurements, cyclic voltammetry (CV) scans across the same concentration ranges revealed no electrochemical response in the case of both COFs. The curves showed no notable changes in peak current nor the emergence of new redox peaks (Figures S24b,c), indicating that the COFs do not directly catalyze glucose oxidation. Instead, the adsorption of glucose molecules at the surface of the COF-coated electrode induces a measurable shift in surface impedance, supporting a nonfaradaic sensing mechanism governed by interfacial charge redistribution rather than direct faradic electron transfer.

In this regard, we hypothesized that the observed change in the surface impedance could be due to the interfacial interactions between glucose molecules and the COF-modified electrode surface (Figure 4e). As glucose molecules diffuse through the electroactive medium, they adsorb onto the COF layer, altering the charge distribution at the electrode–electrolyte interface. The adsorption does not involve a Faradaic (redox) process, but rather modulates the electrical double layer through a capacitive effect, as evidenced by the consistent increase in C_dl_ with increasing glucose concentration.

Notably, the strongest adsorption is expected to occur when the electrophilic azine linkage interacts with the central nucleophilic hydroxyl groups of glucose. This interaction is enhanced in TFPAHz owing to its inherent curvature, which facilitates the formation of a spatially confined, electrophilic pocket that directs glucose into an optimal orientation. Such an arrangement maximizes dipolar interactions, resulting in stronger binding and enhanced sensitivity to glucose. In contrast, the planar structure of TtaHz provides a flat binding surface that weakens the interactions, leading to less efficient binding and reduced sensitivity to glucose.

The COF then serves as a tunable molecular interface that selectively interacts with glucose through a noncovalent adsorption mechanism. These interfacial interactions alter the local dielectric environment at the electrode surface, resulting in a measurable increase in C_dl_. In addition, adsorption-induced interfacial crowding is expected to hinder charge carrier mobility, increasing impedance without involving direct electron transfer processes. The dominance of capacitive behavior confirms the nonenzymatic nature of glucose detection in this system. To assess the selectivity of the sensors to glucose, their response was evaluated in the presence of electroactive species and structurally similar saccharides that coexist with glucose in biological fluids and could potentially interfere with the response. The response to ascorbic acid (AA), uric acid (UA), lactic acid (LA), galactose (Gal), and sucrose (Suc) was evaluated separately under the same experimental conditions performed for glucose sensing for both COF-coated electrodes (Figures S27 and S28). None of the tested interferents exhibited a response comparable to that of glucose and no notable linearity was observed across the tested concentration range (Figure 4f, S29 and S30). The TFPAHz-coated electrode exhibits high selectivity toward glucose at concentrations up to 100 nM, as shown in Figure 4g, where the peak current response for glucose is significantly higher than that of other analytes. However, at higher concentrations (≥100 μM), the peak current response shows reduced selectivity, as some interferents caused modest changes in C_dl_ values (Figure S31), which is consistent with the baseline sensitivity of the underlying SPCE (Table S3).^45,46^ The relatively large error bars observed for the nonelectroactive analytes in Figures S29 and S30 are primarily due to low signal-to-noise ratios inherent in the technique. As shown in Figures S27 and S28, the Nyquist plots for these analytes largely overlap across the tested concentration range (100 aM to 1 mM), with semicircle peaks exhibiting minor variations that are not concentration-dependent, showing a response similar to the electrode behavior shown in Figure 3b. Electroactive analytes exhibit similar behavior for the low concentration ranges. However, a more clear response with smaller error bars is observed in the range of [100 nM–1 mM] in AA and [100 μM–1 mM] in UA for TFPAHz-coated electrodes, and [100 μM–1 mM] in both AA and UA for TtaHz-coated electrodes, reflecting higher signal-to-noise ratios and more distinct impedance changes.

Despite these responses, glucose induced a significantly greater increase in C_dl_, reinforcing the sensor’s selective recognition of glucose over other coexisting species. This distinct response highlights the potential for accurate glucose monitoring in complex biological fluids. The interference from other analytes was minimal for TFPAHz (Figure 4h), with selectivity indices for Gal, AA, LA, Suc, and UA ranging from 9.28% to 33.37% (Figure S32). This suggests that TFPAHz-coated electrode responds strongly to glucose than to other potentially interfering species, which is essential for obtaining a reliable performance in real biological or clinical samples in which interfering compounds are often present. In contrast, TtaHz-coated electrodes displayed a broader response spectrum, with ascorbic acid exhibiting the highest interference, which indicates that this structure might be less suitable for the selective detection of glucose in complex matrices. The applicability of the TFPAHz-coated electrode in complex matrices was further assessed in raw fetal bovine serum (FBS). The electrochemical response of FBS resulted in a C_dl_ value equivalent to that of DPBS solution containing similar glucose concentration with a small marginal deviation (Figure 4i). This comparable response indicates that the TFPAHz-coated electrode maintained excellent glucose sensitivity in complex biological fluid and effectively discriminated against the targeted analyte in the presence of abundant electroactive and adsorptive species, which demonstrates its potential for use in real biological samples without the need for extensive preprocessing.

In addition to testing selectivity against common physiologically relevant interferents, the sensor was further evaluated in artificial sweat samples spiked with glucose (1–1000 nM, *n* = 3) to account for matrix complexity. The tested concentration range is consistent with physiologically relevant sweat glucose levels.^47^ The results shown in Figure S35, confirm the ability of the sensor to function effectively in this range in complex biological media. While the sensor can attain sensitivity to the subattomolar level in clean sample conditions, its capability is significantly degraded in complex sample environments such as FBS and artificial sweat due to matrix effects. Complex biological matrices contain diverse components such as proteins, salts, metabolites, and other small molecules that can induce signal suppression, and elevated biological noise, affecting the detection limit compared to pristine environments.^48,49^

The long-term stability of the azine-linked COFs was evaluated through complementary experiments. First, the samples were incubated for 7 days in polar solvents (DMF and EtOH), aqueous solutions (pH 4 and pH 10), and physiologically relevant media (water and PBS at 37 °C). After incubation, the solids were collected and dried at 90 °C for 24 h prior to further analysis. The PXRD, SEM, and TEM confirm that both COFs retained their crystallinity and morphology under all tested conditions (Figures S36–S40). Functional stability was further evaluated by redepositing the samples onto SPCEs for glucose sensing. The C_dl_ values remained stable, deviating by <5% for TFPAHz and <10% for TtaHz relative to their original values in IPA (Figure S41). Second, TFPAHz-coated electrodes were immersed in FBS at 37 °C for 72 h (Figure S42). The average C_dl_ values fluctuated within ±6% of the initial measurement during the first 48 h and increased to 9.4% after 72 h, possibly due to structural swelling at the interface.^50^ These results confirm that the sensor retains its functional performance in complex biological media for up to 72 h.

The cytotoxicity of materials was assessed using fibroblast cells exposed to increasing concentrations (0, 1, 10, 50, and 150 μg/mL) of both COFs. As shown in Figure S43, TFPAHz had a less pronounced dose-dependent cytotoxicity profile, maintaining ∼80–70% viability, whereas TtaHz had a moderately decreased cell viability with increasing concentration, showing ∼85–60% viability compared to the untreated control. These results indicate that both COFs are tolerated by fibroblasts, while higher doses led to a moderate decrease in cell viability. We further evaluated their suitability for wearable sensing applications by integrating them into a hydrogel-based platform (Figure S44).^51^ When hydrogel substrates are coated with TFPAHz and TtaHz dispersions, a uniform and stable surface coverage is obtained, showing the practical potential of our material to be integrated into next-generation healthcare monitoring technologies.

### 2.4 Theoretical Calculations

The simulation results show that TFPAHz and TtaHz structures adopt a canonical hexagonal P6 symmetry, featuring monolayer lattice parameters of 24.81 and 29.42 Å, respectively. Both structures exhibit preferential eclipsed (AA) stacking configurations and are typically consistent with π–π stacking interlayer distances of 3.64 and 3.32 Å, respectively, in excellent agreement with the experimental values in Figure 1. The left panel of Figure 5a shows the DFT + U computed band structure for the TFPAHz crystal bulk along the k-path Γ → K → M → Γ, connecting the most representative symmetry points, with a resulting band gap at Γ-point of 2.01 eV, showing a moderate band dispersion for the band-states closest to the Fermi energy. Additionally, the computed band gap for TtaHz results in 2.37 eV (Figure S45). These calculated values of 2.01 and 2.37 eV agree with the experimental observation trend, where values of 2.32 and 2.74 eV have been extracted, respectively, from the UV–vis diffuse reflectance spectra.

**Figure 5:**
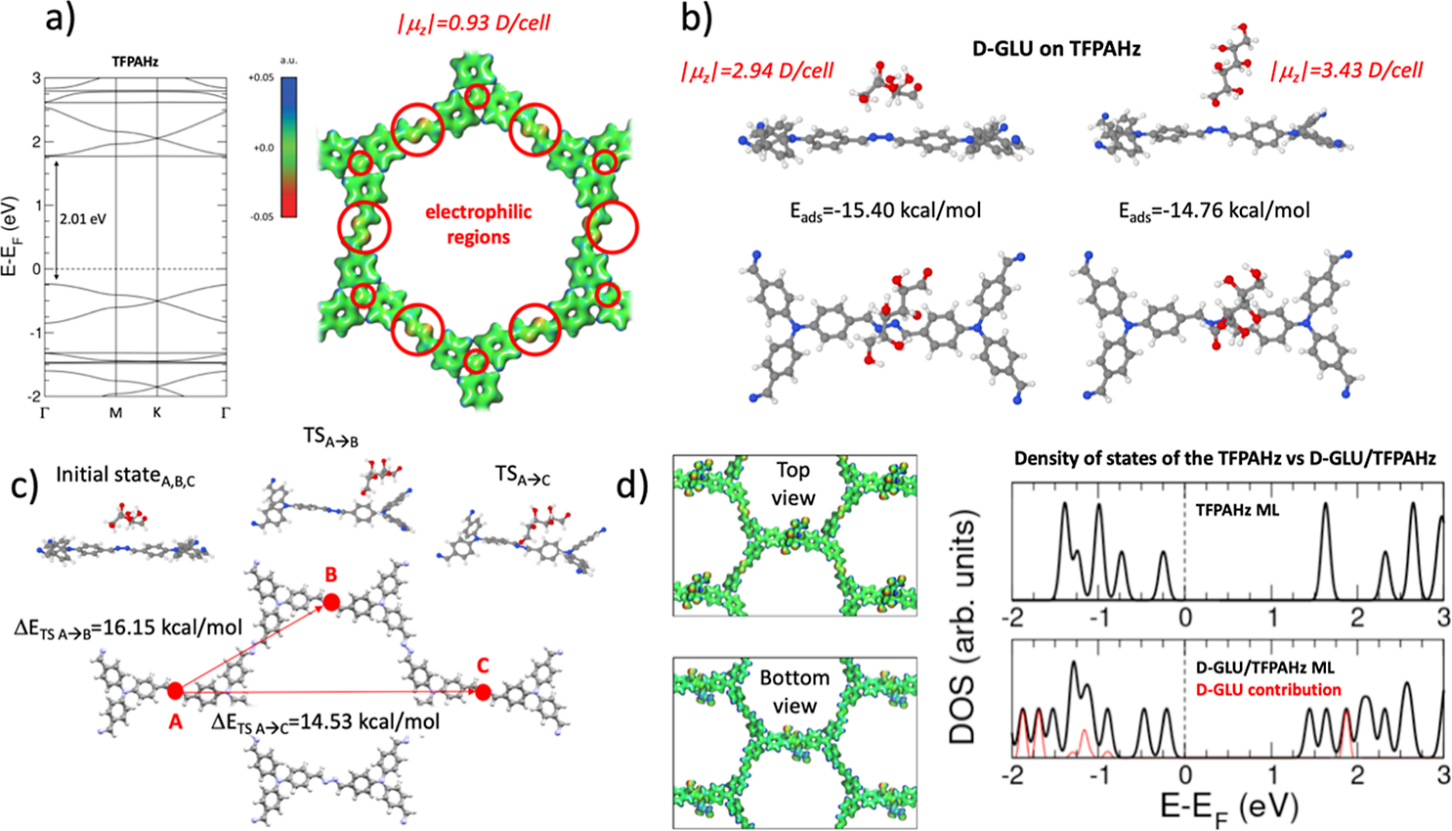
(a) (left) DFT + U computed band structure for TPFAHz crystal bulk (band gap of 2.01 eV at Γ point is indicated superimposed). (right) Electrostatic potential 3D colored-map isosurface for the TFPAHz layer, where the most electrophilic regions are indicated by red circles and the out-of-plane dipole moment value of 0.93 D per unit cell. (b) DFT-optimized most stable adsorption configurations of d-glucose in interaction with the TFPAHz surface, with molecular adsorption energies of −15.40 and −14.76 kcal/mol, and out-of-plane dipole moments of 2.94 and 3.43 D per unit cell, respectively. (c) Transition states resulting from the CI-NEB computation of the minimum energy paths (MEP) for the d-glucose upon TFPAHz on-surface diffusion for a molecule migrating from the most stable adsorption configuration toward its equivalent one in a neighboring cell along two different pathways (A → B and A → C). Transition state barriers of 16.15 and 14.53 kcal/mol are indicated for each MEP. (d) (left) Top and bottom views of the electrostatic potential 3D colored-map isosurface for the most stable d-glucose/TPFAHz adsorption configuration. (right) A comparison between the density of state profiles of the pristine TFPAHz monolayer and the most stable d-glucose/TPFAHz adsorption configuration is indicated later in the molecular contribution.

The right panel of Figure 5a shows the computed electrostatic potential 3D colored-map isosurface for TFPAHz. The most electrophilic regions within the structure correspond to the azine linkages, while the nitrogen atoms of the TFPA moieties exhibit a comparatively lower electrophilic character. These regions are particularly interesting, as they are key in stabilizing donor d-glucose molecules through dipolar interactions.

To elucidate the d-glucose/TFPAHz and d-glucose/TtaHz most stable adsorption configurations, interaction characters, and preferential on-surface molecular positioning and orientation, we have investigated a large variety of starting configurations of d-glucose molecule (one molecule per canonical unit cell) on TFPAHz and TtaHz monolayers. The result of these calculations yields, in all cases tested, the d-glucose exhibiting a stronger interaction with the TFPAHz than with the TtaHz, with a difference in adsorption energies between the most stable d-glucose/TFPAHz and d-glucose/TtaHz adsorption configurations of 7.1 kcal/mol, favorable to the TFPAHz system. Figure 5b shows the two DFT-optimized most stable adsorption configurations of d-glucose in interaction with the TFPAHz surface, with molecular adsorption energies of −15.40 and −14.76 kcal/mol, and out-of-plane dipole moments of 2.94 and 3.43 D per unit cell, respectively. The six most stable d-glucose/TFPAHz and d-glucose/TtaHz adsorption configurations and corresponding adsorption energies are shown in Figures S46 and S47. It is worth to notice the dipolar nature of the d-glucose interaction with the systems. According to the Electrostatic potential 3D colored-map isosurface of Figure 5a for the TFPAHz layer, the most electrophilic region is located in the azine linkages, which is precisely the preferential adsorption surface site to stabilize an electron donor molecule such as the d-glucose (both cases in Figure 5b). In particular, the higher interaction is produced when the azine linkage interacts with the molecular central nucleophilic OH groups with characteristic distances between 2.6 and 2.7 Å (left panel of Figure 5d). Although with a weaker interaction, as explained, a similar interaction behavior can be observed in the case of the d-glucose on the TtaHz system. This finding excellently agrees with the experimentally observed significantly higher d-glucose sensitivity exhibited by the TFPAHz compared to TtaHz.

On the other hand, interestingly, the out-of-plane dipole moment manifests a net increase of around 2 D upon d-glucose adsorption on TFPAHz in its most stable adsorption configuration as a result of the net dipole moment introduced by the molecule in that configuration (around 0.5 D), and the adsorption-induced increased surface dipole moment coming from the slight structural modifications of the surface upon molecular adsorption (around 1.5 D). It is worth mentioning that for the TtaHz case, there is also an increase in the dipole moment upon d-glucose adsorption, but much less significant, around 0.5 D (coming exclusively from the molecular dipole moment). This theoretical prediction for the d-glucose/TFPAHz system translates into a non-negligible charge rearrangement, pointing out a change in the surface properties, which, as experimentally observed, leads to measurable changes in the surface impedance.

Climbing-Image Nudged Elastic Band (CI-NEB) method was employed to determine the minimum energy paths (MEP) and activation barriers for d-glucose diffusion on TFPAHz surface, analyzing the molecular migration from an equilibrium adsorption site to an equivalent neighboring position. This approach fully relaxed the initial, final, and 20 intermediate image states. The computed transition states and energy barriers corresponding to d-glucose migrating from its most stable adsorption configuration along two distinct diffusion pathways (A → B and A → C) within the periodic lattice (Figure 5c). Calculated energy barriers result in 16.15 and 14.53 kcal/mol for each of A → B and A → C MEP, respectively, consistent with a certain on-surface diffusion at 300 K according to the Boltzmann statistics. Nonetheless, at this temperature, the thermal energy *k*_B_ *T* = 0.6 kcal/mol will allow enough molecular residence time on the surface to modify the electronic surface properties, translating into measurable surface impedance changes.

All the above-mentioned theoretical predictions strongly indicate a significant modification of the electronic properties of the surface upon d-glucose adsorption, particularly on the TFPAHz surface. To substantiate these findings and directly assess the impact of molecular adsorption on surface properties, the density of states (DOS) profiles were computed for both the pristine TFPAHz monolayer and the d-glucose/TFPAHz interfacial system in its most stable adsorption configuration. The results, presented in the right panel of Figure 5d, reveal that the DOS profile of the TFPAHz monolayer closely resembles the electronic structure of the bulk TFPAHz (Figure 5a), with an expected slight band gap reduction to 1.85 eV due to the absence of π–π stacking interactions in the monolayer.

Upon d-glucose adsorption, distinct modifications in the electronic structure can be observed. Notably, the band gap decreases from 1.85 to 1.6 eV, attributed to two key factors: (i) the disruption of the 2-fold degeneracy in the conduction band states due to adsorption-induced symmetry perturbations, resulting in a splitting of the conduction band peak, and (ii) an increase in the work function of the system (defined as the energy difference between the Fermi level and the vacuum level) by 0.33 eV, driven by the enhanced surface dipolar moment upon molecular adsorption. This work function shift increases the interfacial resistance by impeding electron transport.

Additionally, a significant accumulation of electronic states below the Fermi level is observed, arising from a “push-up” effect of occupied states contributed by the d-glucose molecule. In contrast, the electronic structure of TtaHz remains largely unaffected upon glucose adsorption (Figure S48). This distinct electronic response highlights the superior sensitivity of the TFPAHz surface to d-glucose, positioning it as a promising platform for molecular sensing applications.

To directly link the theoretical dipole moment change with the experimental impedance response, we note that glucose adsorption induces a significant increase in the out-of-plane dipole moment of the TFPAHz surface (∼2 D per unit cell). This enhanced dipolar polarization perturbs the electrochemical double layer (EDL) at the electrode–electrolyte interface, resulting in measurable changes in the double-layer capacitance (C_dl_) as observed by EIS. The induced dipole moment and associated surface charge redistribution increase the local dielectric constant and the interfacial resistance, consistent with the observed non-Faradaic, capacitive sensing response. This provides a mechanistic rationale for the excellent correlation between molecular adsorption and impedance signal transduction (Figure 6).

**Figure 6:**
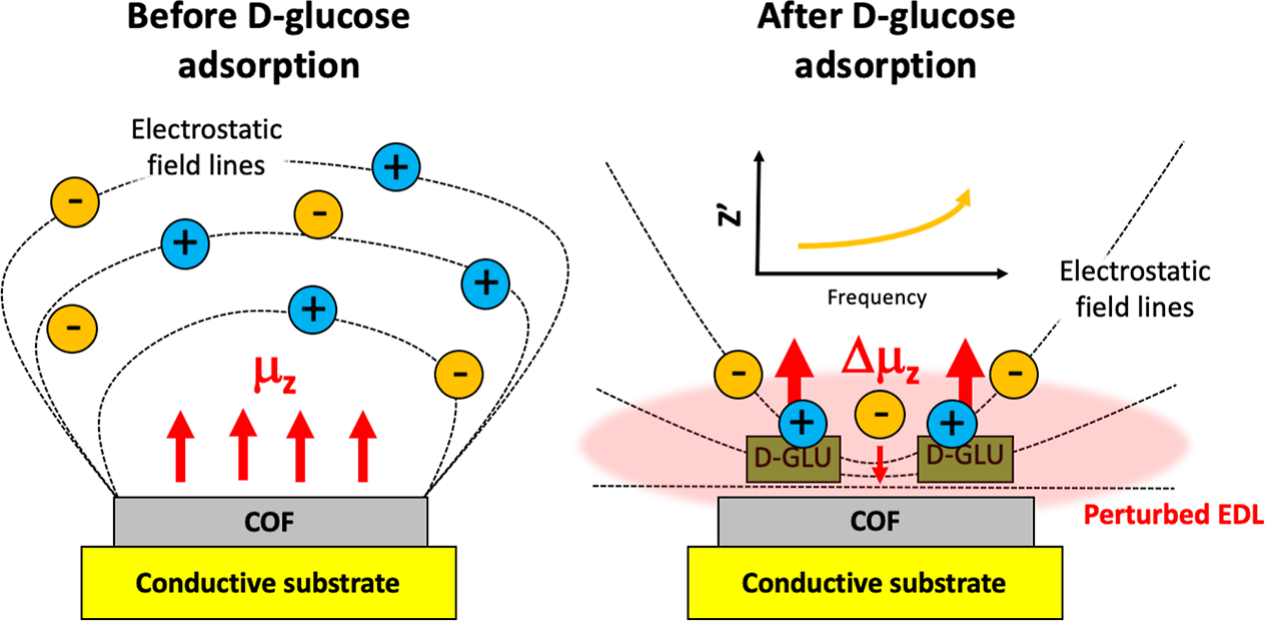
Schematic illustration of the proposed sensing mechanism showing the interfacial configuration before (left) and after (right) d-glucose adsorption on the COF surface. Prior to adsorption, the electrostatic field lines and electric double layer (EDL) are weakly perturbed, with a baseline dipole moment (μ_*z*_). Upon d-glucose adsorption, the interfacial dipole (Δμ_*z*_) increases due to charge redistribution, leading to a pronounced perturbation of the EDL and modification of the interfacial capacitance. This change is transduced as a measurable variation in impedance (*Z*′), as illustrated in the inset plot.

To rationalize the experimentally observed selectivity for glucose, we performed comparative DFT calculations for fructose, ascorbic acid, and uric acid (Figure S49). These molecules were chosen for their structural or functional relevance. Fructose is a structural isomer of glucose and is functionally similar, serving as a common dietary sugar and metabolic substrate. Ascorbic acid, a redox-active antioxidant, and uric acid, a metabolic byproduct with aromatic and polar functionalities, are both present in biological samples and known to interfere with glucose detection systems due to their electrochemical activity and adsorption capacity. For each analyte, we calculated both the adsorption energy and the induced out-of-plane dipole moment resulting from the interaction with the TFPAHz surface. Glucose exhibited the strongest binding affinity (−15.40 kcal/mol) and induced the most significant surface dipole moment (−2.94 D/cell), whereas fructose (−10.30 kcal/mol, −1.75 D/cell), ascorbic acid (−7.46 kcal/mol, −1.42 D/cell), and uric acid (−10.68 kcal/mol, −1.61 D/cell) showed considerably lower interaction energies and dipolar effects.

These variations in adsorption strength and dipolar perturbation directly influence the sensor response. The change in electrochemical impedance arises from the disruption of the electrical double layer (EDL) caused by adsorption-induced dipole changes. Glucose, inducing the most significant local polarization, produces the most pronounced shift in interfacial capacitance and consequently a strong impedance signal. In contrast, the other analytes cause weaker interfacial perturbations, resulting in minimal or negligible changes in impedance. These theoretical analyses highlight that both a strong binding affinity and significant dipolar polarization govern the selectivity mechanism of the COF-based sensor.

## 3 Conclusion

This study presents a structure-driven strategy to enhance nonenzymatic electrochemical glucose sensing. Through a strategic replacement of the central triazine ring with a nitrogen atom, we introduced conformational flexibility to the C_3_ symmetric building unit and curvature to the framework structure, both of which enhanced glucose–framework interactions and interfacial charge dynamics. This rational molecular design endowed the system with exceptional sensing performance, achieving attomolar-level sensitivity, broad dynamic range [1 aM–5 mM], and pronounced selectivity against physiologically relevant interferents, including ascorbic acid, uric acid, lactic acid, galactose, and sucrose. The sensing mechanism is governed by nonfaradaic surface interactions, enabling signal transduction without requiring redox-active mediators. Notably, the demonstrated stability, reproducibility, and responsiveness of these COF-based sensors highlight their potential for integration into wearable and implantable diagnostic platforms. To obtain an in-depth mechanistic understanding of glucose sensing performance, we combined experimental observations with theoretical calculations. Particular attention was given to how molecular design governs electronic structure, surface interaction energetics, and dipolar perturbations during glucose adsorption. This integrative approach enables a fundamental understanding of the pronounced difference in glucose response between TFPAHz and TtaHz, laying the groundwork for the rational design of next-generation impedimetric sensors. Several improvements to the sensor can focus on miniaturizing the sensor design for integration into point-of-care and wearable devices toward clinical translation. Our COF-based sensor demonstrates excellent selectivity for physiologically relevant interferents under controlled static conditions. However, using the sensor for actual applications requires other considerations that can influence the sensor performance including continuous fluid flow, skin barrier interactions, and the presence of complex matrices like sweat. To bridge this gap between lab-scale validation and practical use, future work can focus on simulating more realistic conditions accounting for such factors to evaluate the reliability and accuracy of the sensor in a more dynamic and skin-relevant environments to provide better insights into the translational potential of these COF platforms for noninvasive glucose monitoring.

## Supplementary Material



## Notes

Materials and instrumentation, synthetic details, characterization, electrochemical measurements, sensing experiments, and theoretical calculations (PDF)
