# Supplementary material for: Tuning Core Flexibility and Curvature in Azine-Linked Covalent Organic Frameworks for Attomolar-Level Impedimetric Sensing of Glucose
Source: J Am Chem Soc. 2025 Oct 8;147(43):39247–58. doi: 10.1021/jacs.5c10578 (PMC12576810; doi:10.1021/jacs.5c10578)
Supplement: Supplementary file 1 [file ja5c10578_si_001.pdf]

Supporting Information

Tuning Core Flexibility and Curvature in Azine-Linked Covalent Organic Frameworks for Attomolar-Level Impedimetric Sensing of Glucose

Nada Elmerhi,[§,††,#] Sara Awni Alkhatib,[||,††,‡‡,#] Sushil Kumar,[§,††] José Ignacio Martínez,[†] Nabila Yasmeen,[||] Najat Maher Aldaqqa,[§] Blaž Belec,[‡] Anna-Maria Pappa,[||,††,‡‡,*] and Dinesh Shetty[§,††,*]

[§]Department of Chemistry, Khalifa University of Science and Technology, Abu Dhabi, PO Box: 127788, United Arab Emirates.

[||]Department of Biomedical Engineering and Biotechnology, Khalifa University of Science and Technology, Abu Dhabi, PO Box: 127788, United Arab Emirates.

[†]Instituto de Ciencia de Materiales de Madrid (ICMM-CSIC). C/Sor Juana Inés de la Cruz 3, 28049 Madrid, Spain.

[‡]Materials Research Laboratory, University of Nova Gorica, Vipavska 11c, 5270 Ajdovscina, Slovenia.

[††]Center for Catalysis and Separations, Khalifa University of Science and Technology, Abu Dhabi, PO Box: 127788, United Arab Emirates.

[‡‡]Biotechnology Center, Khalifa University of Science and Technology, Abu Dhabi, PO Box: 127788, United Arab Emirates

[#]Equal contribution

Section	Content	Page/s
S-1	Materials and Instrumentation	3-5
S-2	Synthetic Procedures and Schemes	5-6
S-3	PXRD	7-8
S-4	FTIR	9
S-5	XPS	10
S-6	SEM	10
S-7	TEM	11
S-8	SEM-EDS	11-12
S-9	Gas adsorption	12-13
S-10	TGA and UV-Vis	13-14
S-11	Electrochemical analyses and sensing	14-38
S-12	Theoretical calculations	39-43
S-13	References	44-46

Section S-1: Materials and Instrumentation

Materials

4,4',4"-(1,3,5-triazine-2,4,6-triyl)tribenzaldehyde (Tta) and tris(4-formylphenyl)amine (TFPA) were purchased from TCI chemicals. Hydrazine hydrate was purchased from Sigma Aldrich. All other chemicals, including p-Toluenesulfonic acid (TCI chemicals), N, N-dimethylacetamide (DMA) (Sigma-Aldrich), and acetone (Merck), were purchased from commercial sources and used without any further purification. The materials used in electrochemical analyses and sensing were purchased from Sigma Aldrich, Germany, and were analytical grades and adhered to high purity standards; D-(+)-glucose (≥99.5%), isopropanol (2-propanol), and absolute ethanol for analysis. Gibco Fetal Bovine Serum (FBS, containing 5 mg/dl glucose), Dulbecco's Phosphate Buffered Saline (DPBS) (1X), and Potassium Hexacyanoferrate III $K_3[Fe(CN)_6]$ were sourced from Thermofisher, UK. $K_3[Fe(CN)_6]$ served as an electrolyte throughout the experimental procedures for EIS sensing experiments ensuring consistency in results. Screen printed carbon electrodes (SPCE) were purchased from Metrohm DropSens (DRP-110). Deionized water (DI) water was used to wash electrode surfaces.

Instruments

Powder X-ray diffraction (PXRD): The PXRD patterns of the samples were analyzed using a PANalytical Empyrean XRD equipped with a CuKα radiation source, operating at 40 kV and 45 mA. Patterns were collected over a 2θ range of 2.5° to 50° with a step size of 0.01°. Data processing and background correction were performed using X'pert High Score Plus software.

Fourier transform infrared (FTIR): The FTIR spectra were collected using a Bruker Optics ALPHA-E spectrophotometer with a universal Zn-Se ATR (attenuated total reflection) or Diamond ATR (Golden Gate) accessory in the 600-4000 cm^{-1} region at 4 cm^{-1} resolution with 24 scan rate.

Nuclear Magnetic Resonance Spectroscopy (NMR): Solid state ^{13}carbon cross-polarization magic angle spinning (CP/MAS) ^{13}C NMR spectra were acquired using a Bruker Avance 500 Wide Bore (500 MHz) NMR spectrometer with a magic angle spinning rate of 18.0 kHz,

operating at an ambient temperature with a static field of 14.1 T. The obtained NMR data were processed using Top Spin 4.1.4 software.

X-ray photoelectron spectroscopy (XPS): XPS analyses were performed using a Thermo Fisher Scientific Escalab Xi+ spectrometer equipped with Al Kα X-ray source and a monochromator with a take-off angle of 90°. The charge neutralizer was turned on during the measurements. The samples were placed over a silicon wafer attached to carbon tape. Binding energy scale correction was done based on C-C/C-H peak at 284.8 eV in the C 1s spectra.

Scanning electron microscopy (SEM): SEM images were obtained using a field-emission scanning electron microscope (FESEM, JEOL JSM-7100f TTLS) equipped with an energy-dispersive X-ray spectrometer (EDXS, Oxford X-Max80). A ~10 μL aliquot of the dispersed sample in aqueous ethanol was drop-cast onto a clean silicon substrate and dried prior to imaging.

Transmission electron microscopy (TEM): TEM images were obtained using a field-emission electron microscope (JEOL JEM-2100UHR, Tokyo, Japan), operating at 200 kV and equipped with an energy-dispersive X-ray spectrometer (EDXS, Oxford X-Max80T). The sample was prepared by drop-casting a ~5 μL of the aqueous dispersion over a copper grid-supported lacy carbon film.

Gas adsorption: Surface area and porosity analyses were performed using the Autosorb iQ instrument by Anton Paar. A weighed amount of the sample (~ 20-30 mg) was activated by degassing at 120 °C for 10 h, followed by backfilling with N_2 gas. Adsorption-desorption isotherms were then recorded under incremental exposure to ultrahigh-purity nitrogen up to 1 atm (maintained in a liquid nitrogen bath (77 K)). The Brunauer-Emmett-Teller (BET) method was employed to calculate the specific surface area from the adsorption data using the instrument software. The pore size distribution was calculated using the non-local density functional theory (NLDFT) method.

Thermogravimetric analysis (TGA): TGA was performed using Perkin-Elmer Simultaneous Thermal analyzer STA 6000 under N_2 atmosphere at a heating rate of 10 ºC min^{-1} within a temperature range of 30-900 °C.

Solid-state Ultraviolet-Visible (UV-vis) spectroscopy: The LAMBDA 1050 UV/Vis/NIR spectrometer was used to analyze the diffuse reflectance spectra over the wavelength range of 200–1000 nm. The obtained spectra were converted to Kubelka-Munk (F(R) vs. energy) plots, and the band gaps were determined using the Tauc plot method.

Section S-2: Synthetic Procedures and Schemes

Synthesis of TtaHz. A mixture of 4,4',4"-(1,3,5-triazine-2,4,6-triyl)tribenzaldehyde (Tta) (100.00 mg, 254.19 μmol) and p-toluenesulphonic acid (PTSA) (218.86 mg, 1.27 mmol) was ground for 5 min. A solution of hydrazine hydrate (Hz) (18.27 μL, 381.29 μmol) was added to the PTSA-amine paste and mechano-mixed thoroughly for 10 min. The reaction was heated in a closed vial for 24 h at 90 °C. The resulting solid was washed with water, DMA, and acetone to remove any impurities and unreacted monomers, followed by drying at 90 °C for 24 h. The dried TtaHz was used for further characterization and measurements.

Synthesis of TFPAHz. A mixture of tris(4-formylphenyl)amine (TFPA) (100.00 mg, 303.62 μmol) and p-toluenesulphonic acid (PTSA) (261.42 mg, 1.52 mmol) was ground for 5 min. A solution of hydrazine hydrate (Hz) (21.82 μL, 455.44 μmol) was added to the PTSA-amine paste and mechano-mixed thoroughly for 10 min. The reaction was heated in a closed vial for 24 h at 90 °C. The resulting solid was washed with water, DMA, and acetone to remove any impurities and unreacted monomers, followed by drying at 90 °C for 24 h. The dried TFPAHz was used for further characterization and measurements.

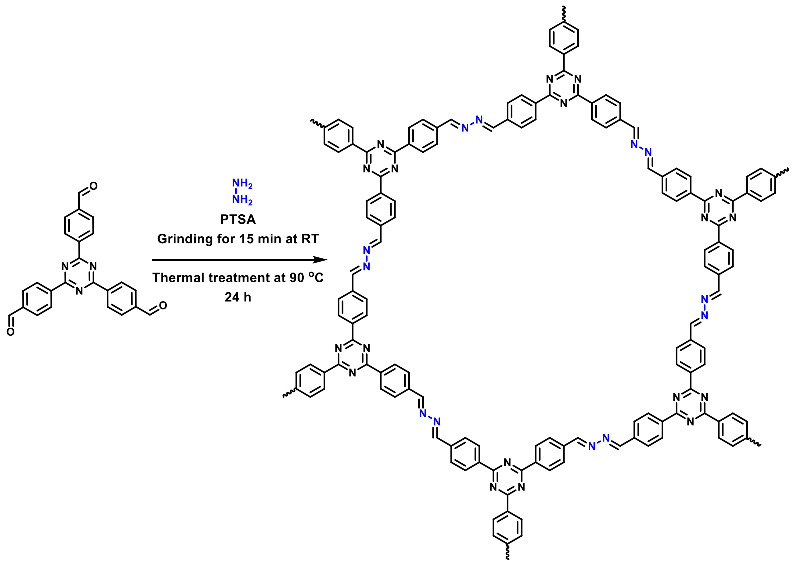

Figure S1. Synthetic scheme of TtaHz.

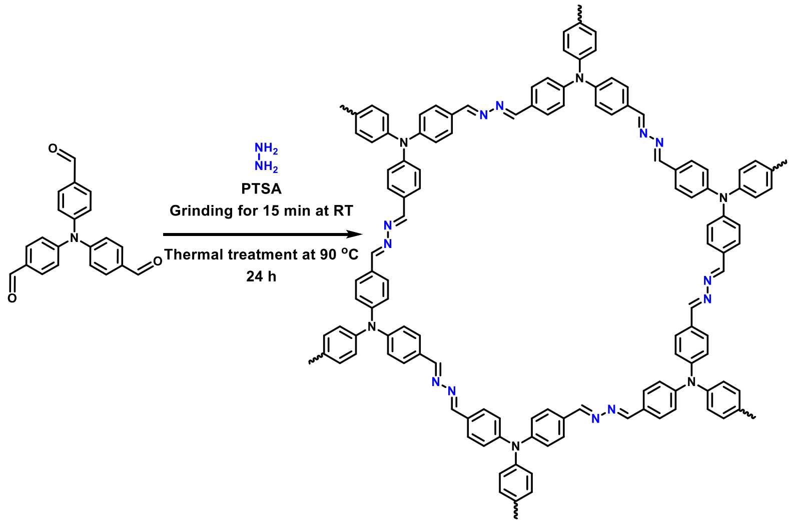

Figure S2. Synthetic scheme of TFPAHz.

Section S-3: PXRD

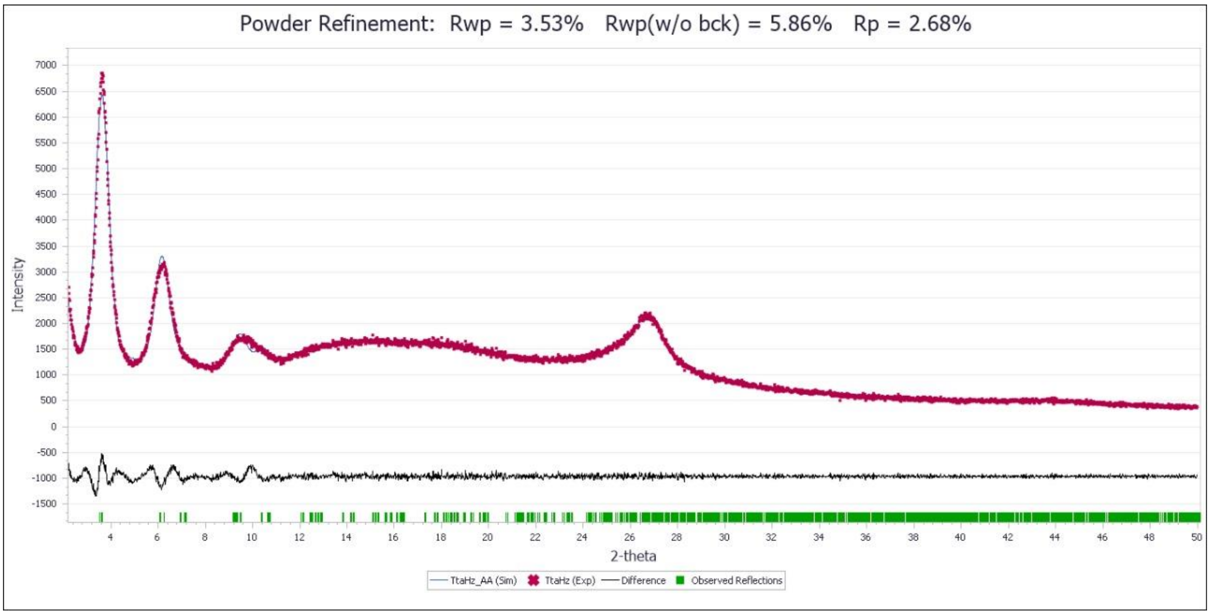

Figure S3. Comparison of experimental PXRD pattern of TtaHz with corresponding simulated pattern and Pawley refined difference. The inset displays Rwp, and Rwp (w/o bck), and Rp (lattice parameters: R, weighted profile R values: Rwp, Un-weighted profile R values Rp).

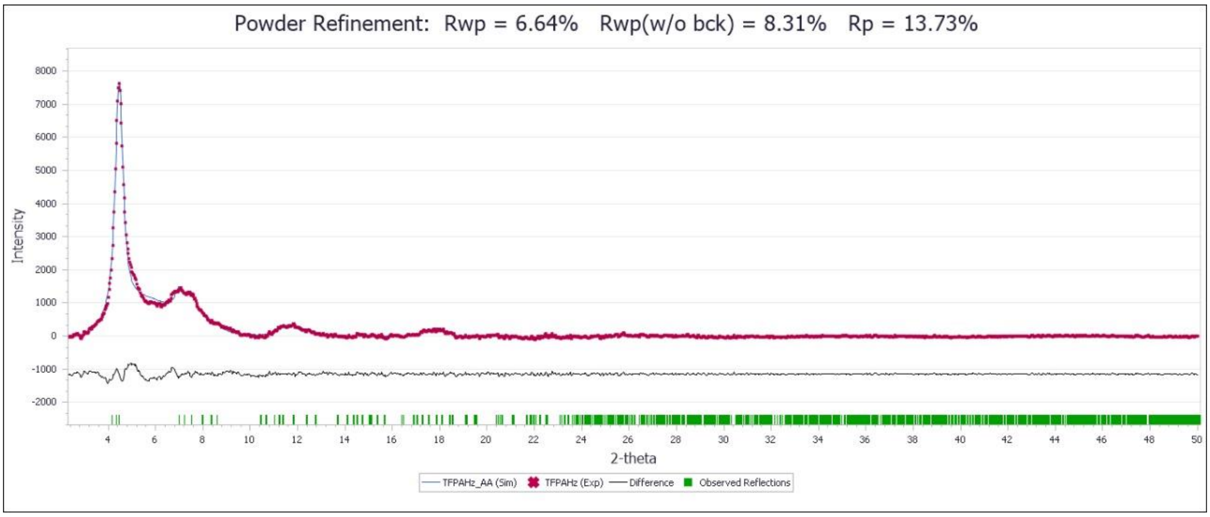

Figure S4. Comparison of experimental PXRD pattern of TFPAHz with corresponding simulated pattern and Pawley refined difference. The inset displays Rwp, and Rwp (w/o bck), and Rp (lattice parameters: R, weighted profile R values: Rwp, Un-weighted profile R values Rp).

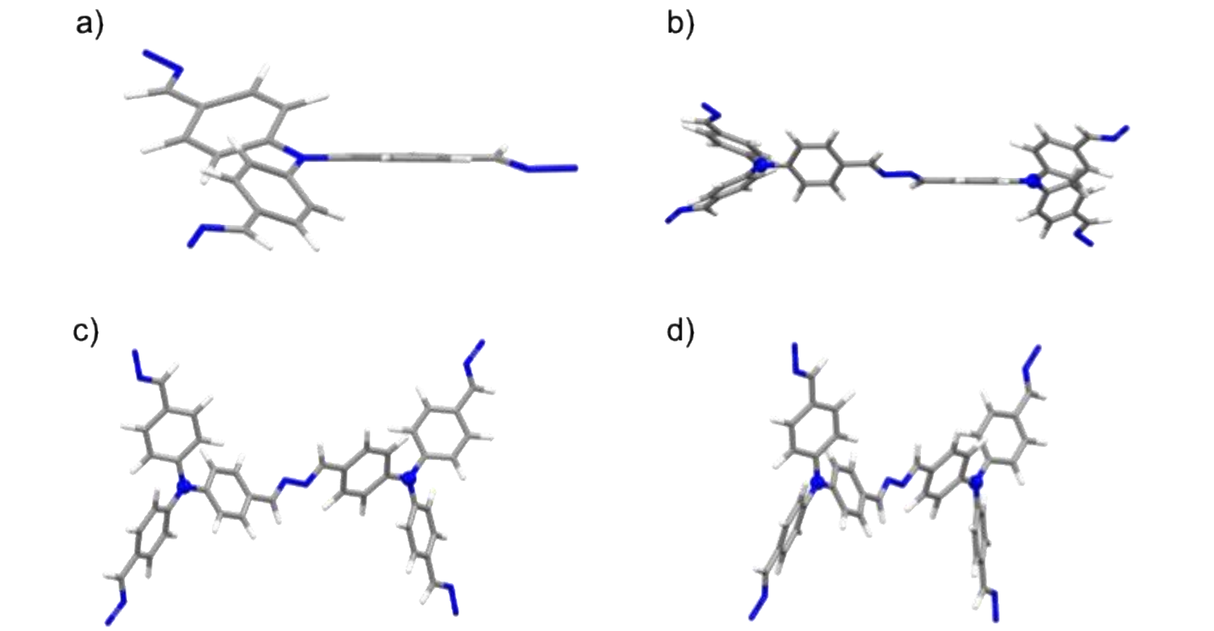

Figure S5. Structural views of TFPAHz rotated to different positions, highlighting its nonplanar and curved structure.

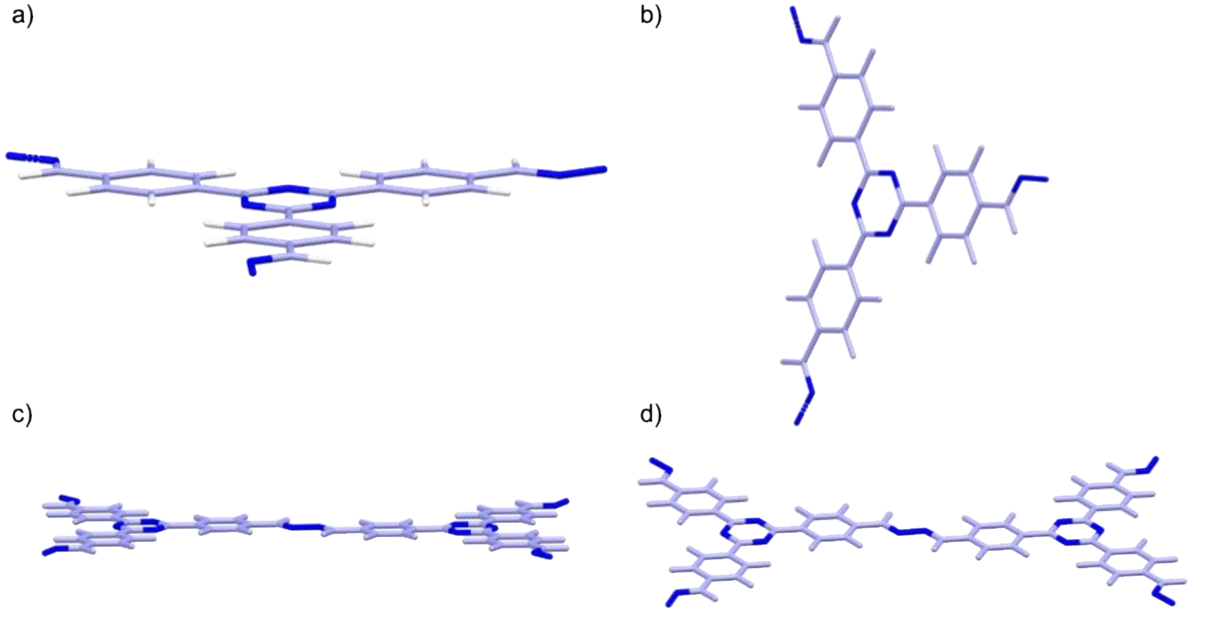

Figure S6. Structural views of TtaHz rotated to different positions, highlighting its planar structure.

Section S-4: FTIR

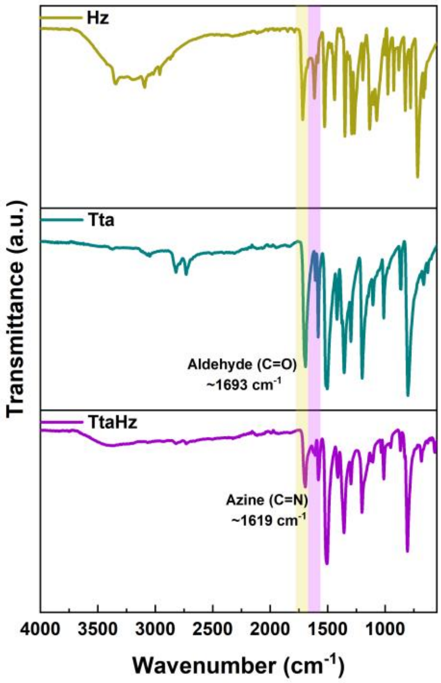

Figure S7. FTIR spectra of TtaHz along with its constituent monomers.

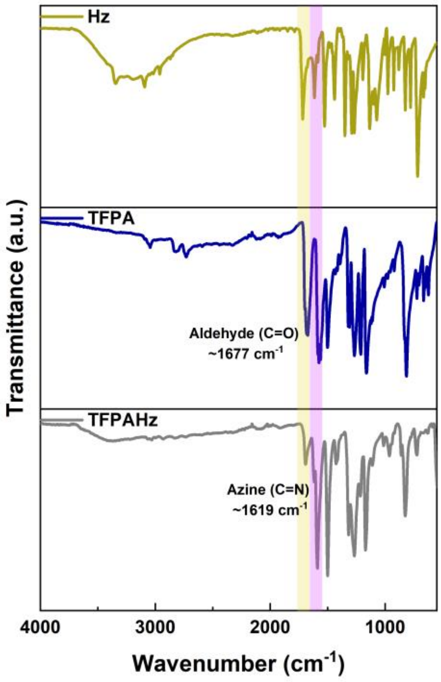

Figure S8. FTIR spectra of TFPAHz along with its constituent monomers.

Section S-5: XPS

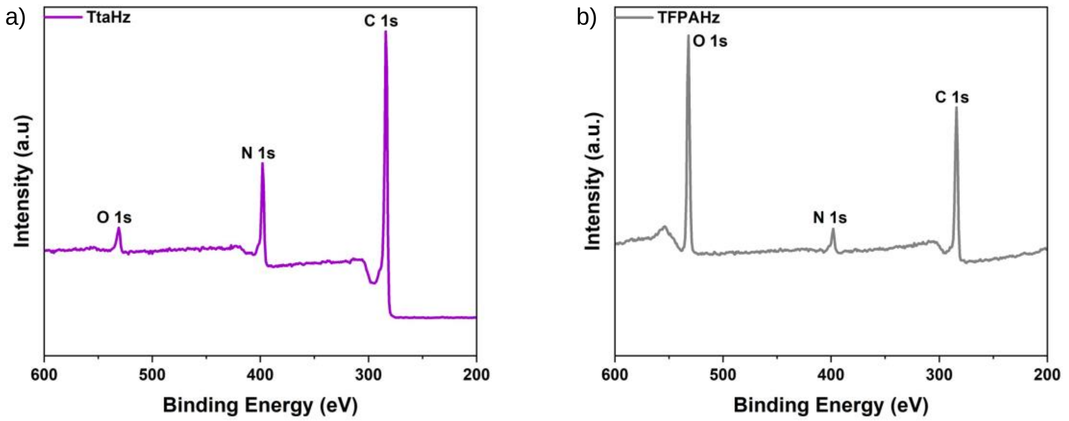

Figure S9. XPS survey spectra of a) TtaHz and b) TFPAHz. The oxygen detected in the XPS survey spectra of both COFs likely originated from trapped residual solvents (e.g. water, DMA, and acetone) used during the washing process.

Section S-6: SEM

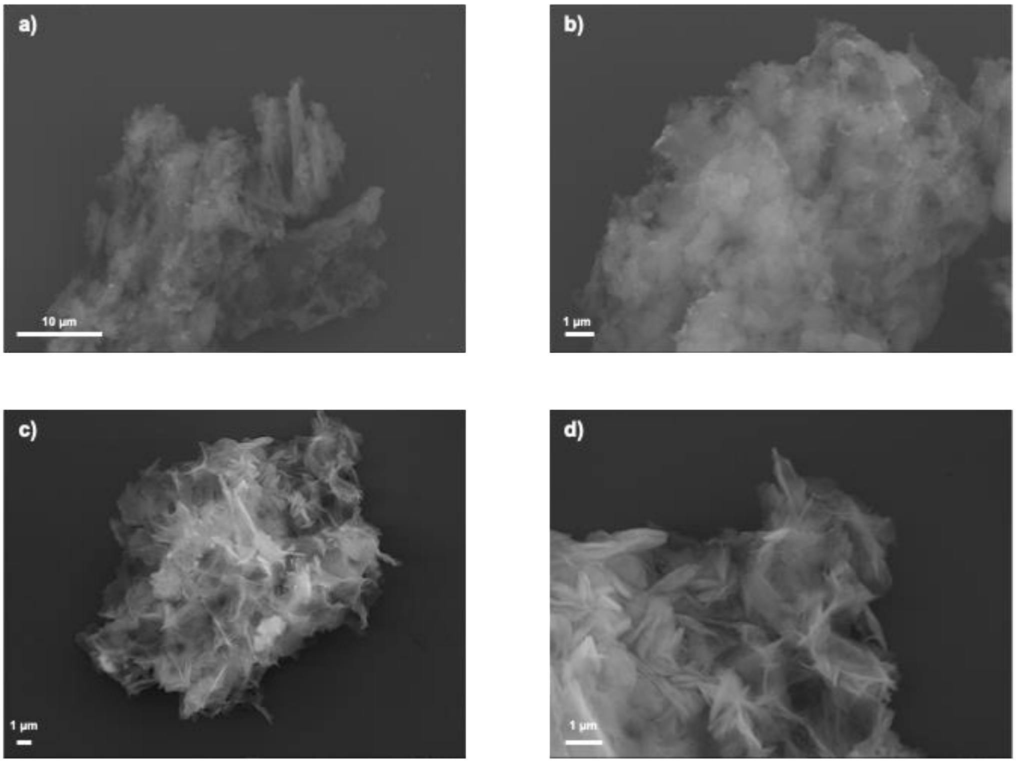

Figure S10. SEM images of a-b) TtaHz and c-d) TFPAHz.

Section S-7: TEM

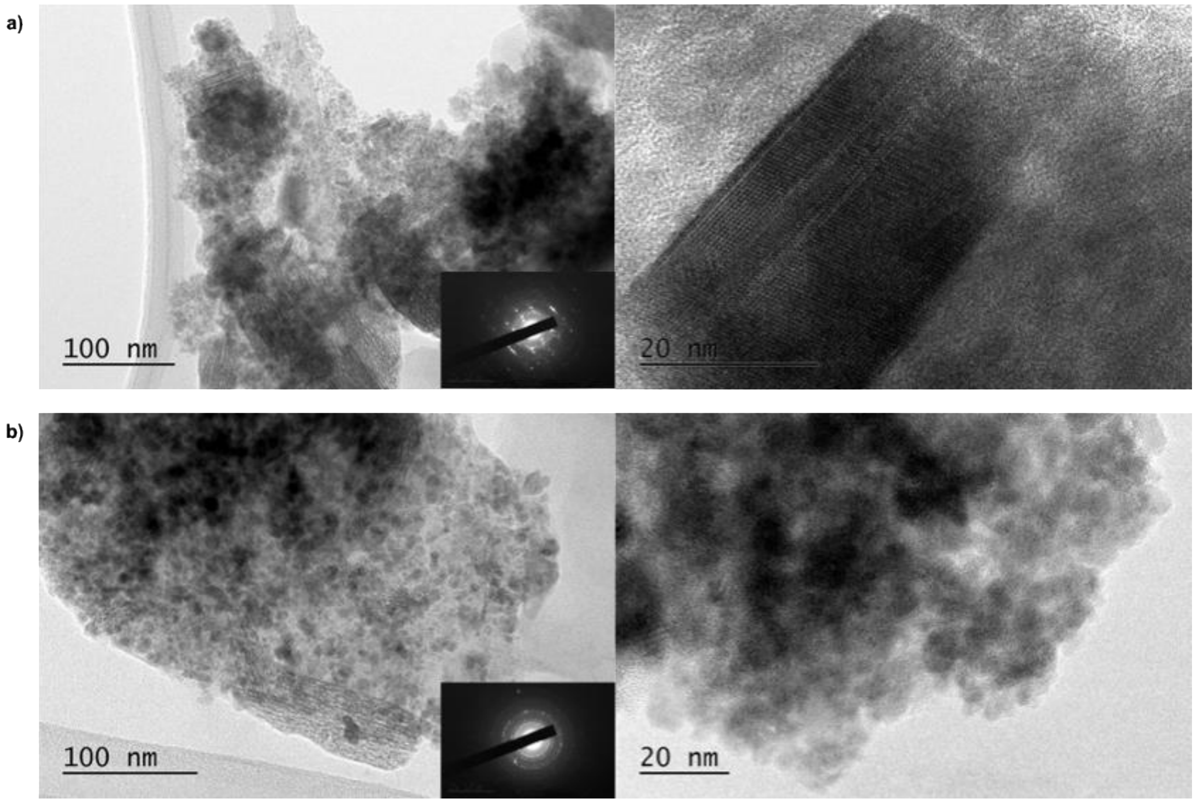

Figure S11. TEM images of a) TtaHz and b) TFPAHz at different magnifications, along with their selected area electron diffraction (SAED) patterns (insets). The SAED patterns reveal the presence of nanocrystalline domains within the COFs, confirming their crystallinity.

Section S-8: SEM-EDS

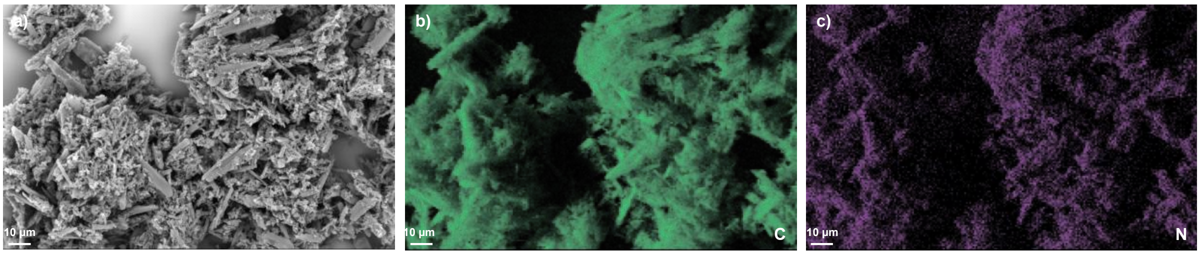

Figure S12. SEM elemental mapping of TtaHz.

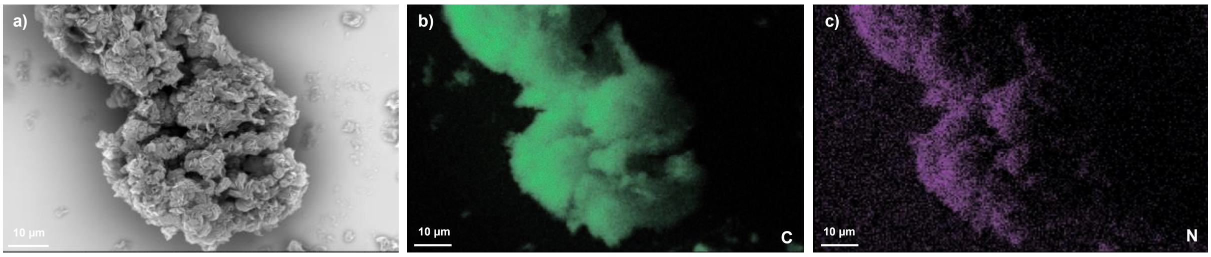

Figure S13. SEM elemental mapping of TFPAHz.

Table S1. Elemental composition (weight percentage) of TtaHz and TFPAHz determined by SEM-EDS.

**Material**	**Elements**	
-	Carbon	Nitrogen
TtaHz	85.784	14.216
TFPAHz	95.770	4.230

Section S-9: Gas Adsorption

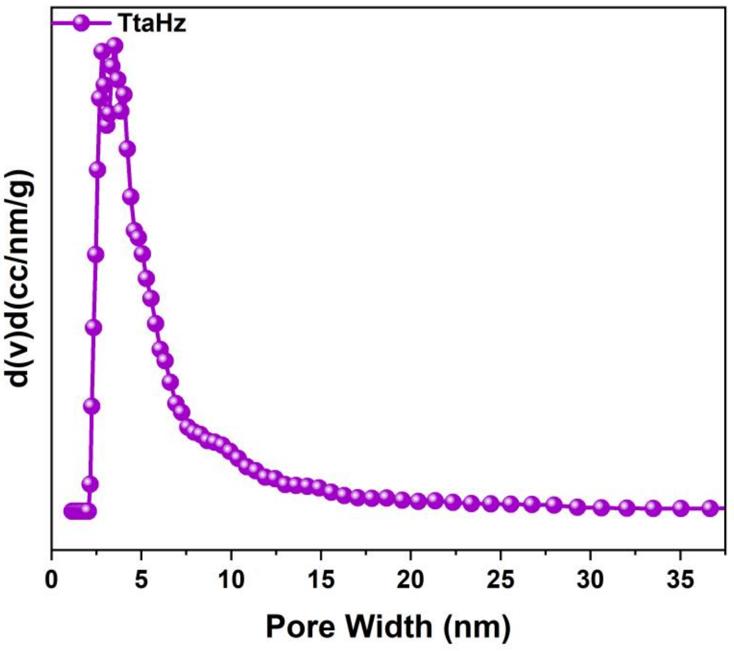

Figure S14. Pore-size distribution profile of TtaHz.

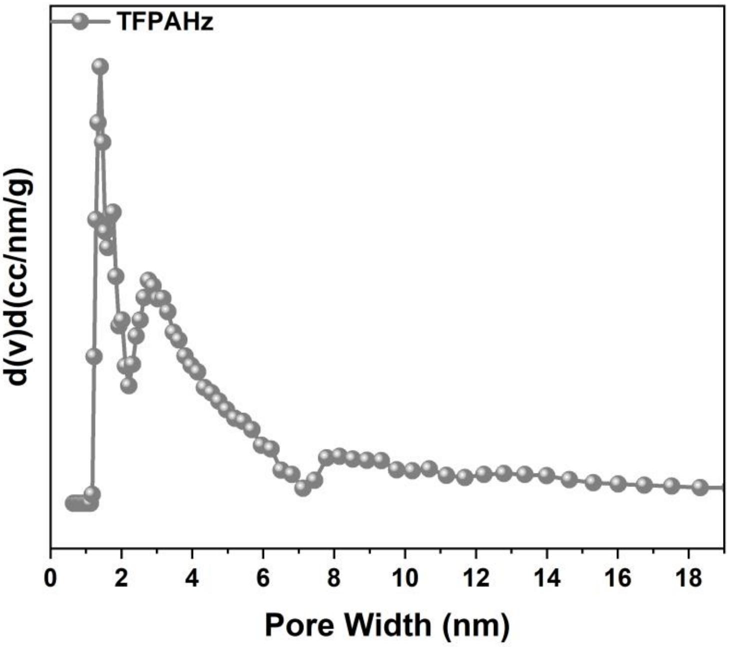

Figure S15. Pore-size distribution profile of TFPAHz.

S-10: TGA and UV-Vis

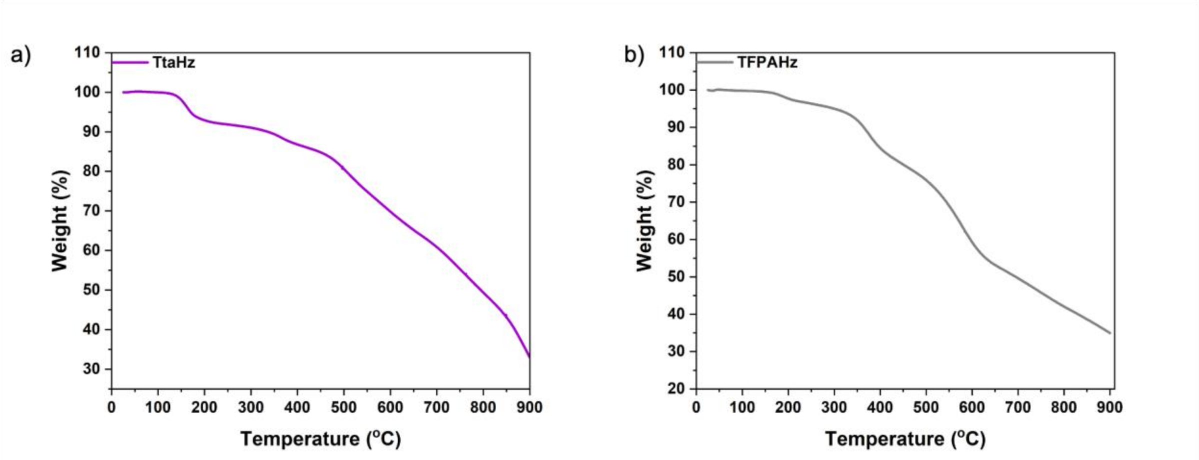

Figure S16. TGA for a) TtaHz and b) TFPAHz.

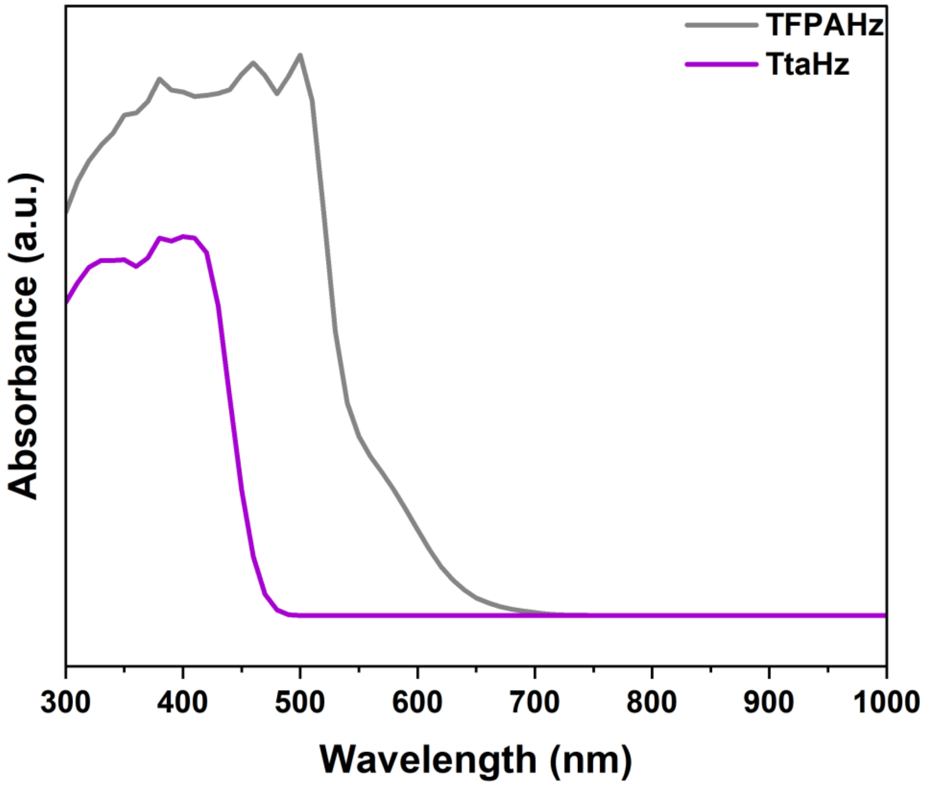

Figure S17. UV-Vis absorption spectra of TtaHz and TFPAHz.

S-11: Electrochemical Analyses and Sensing

Preparation of COF dispersions and COF-coated electrodes

COF dispersion of 1 mg/ml was prepared by dispersing 10 mg of the synthesized COF into 10 ml of 99.5% isopropanol. The dispersion was sonicated for 3 hours using a Digital Pro + Ultrasonic Cleaner to break down any agglomerates and ensure a uniform suspension of COF particles in the dispersion. The solution was used immediately for subsequent coating on screen-printed carbon electrode (SPCE). To prepare the SPCE for coating, the electrode was initially sonicated in 30% ethanol for 10 mins, then rinsed and sonicated for another 10 mins in deionized (DI) water to eliminate any left residues. Then it was dried using compressed air and left to air dry completely before use. A mask exposing only the working electrode (WE) area was placed on SPCE surface before the COF's coating was introduced to WE. Two different techniques for coating were followed and compared, drop-casting and spin coating. Drop casting involved dropping 1 µL of well-dispersed COF on the WE area then left to air

dry for 10 mins. For the spin-coating method, 10 μL of COF dispersion was spin-coated on WE surface using spin-coater Ossila in three different stages: 1500 rpm for 10 seconds, 3000 rpm for 10 seconds, and 500 rpm for 5 seconds. The process was repeated 3 times to ensure the formation of uniform, consistent, and significant coating thickness. The mask was gently removed and the SPCE was allowed to be air dried for 10 mins before subsequent electrochemical measurements.

Electrochemical Measurements

The electrochemical performance of the COF-coated electrodes was assessed using cyclic voltammetry (CV) and electrochemical impedance spectroscopy (EIS). The CV measurements were performed in the potential range from -1.0 V to +1.0 V at a scan rate of 10 mV/s and a current value of 1 μA. The EIS measurements were performed in the frequency range from 100 kHz to 0.1 Hz, with an amplitude of 10 mV around the open circuit potential (OCP). The glucose (Glu) solutions were prepared in PBS X1 for CV measurements and in 5 mM $K_3[Fe(CN)_6]$ for EIS measurements. Each measurement was performed in triplicates to ensure reproducibility and reliability of the data. The measurements were conducted in a Faraday cage to minimize noise and any external interference. The electrochemical measurements were recorded using Autolab Potentiostat/Galvanostat (PGSTAT101) and analyzed using the NOVA 12.1 software.

Selectivity and Interference Studies

The selectivity of the COF-coated electrodes to glucose was assessed against five common electroactive and structurally related interferents typically present in biological fluid: ascorbic acid, lactic acid, uric acid, galactose, and sucrose. The study involved both individual and mixed interferent testing. Each species was tested independently at concentrations of 0 M, 100 aM, 100 fM, 100 pM, 100 nM, 100 μM, and 1 mM using freshly prepared COF-coated electrodes. The resulting electrochemical responses were compared to those recorded for glucose at equivalent concentrations. In addition, sequential interference testing was performed, in which baseline measurements were first recorded on the COF-coated electrodes, followed by the stepwise addition of each interferent at 100 nM, with signal responses collected after each addition. To further validate glucose selectivity, a mixed-interferent experiment was performed using a solution containing 100 nM of each interferent, and the response was compared before and after the introduction of 100 nM glucose into the mixture. Finally, to

assess sensor performance in complex biological media, we evaluated the COF-coated electrodes in raw fetal bovine serum (FBS); 1 µL of FBS was added to the electrolyte containing the redox probe $K_3[Fe(CN)_6]$, and the electrochemical response was recorded.

Artificial sweat testing

Artificial sweat samples was prepared using EN1811:2011 European Standard,[1] which contains sodium chloride, urea, ammonia, lactic acid, potassium chloride, and glucose dissolved in ultrapure water (18.2 MΩ cm) and the pH was adjusted to 7.2 using NaOH (1 M). then the solution was filtered (0.22 µm PES) and used freshly for testing. A 1 mM glucose stock was prepared and diluted into the freshly prepared artificial sweat to obtain the tested concentrations [0.5, 1, 50, 100, 250, 500, 1000] nM. EIS was recorded for each concentration after a 5 min stabilization (n = 3) and blank matrix (artificial sweat without glucose) was run between levels to check for carryover. Between measurements the electrode was rinsed and equilibrated for 5 min in blank artificial sweat and then gently rinsed and transferred to the test solution, and no redox mediator was added.

Cytotoxicity of COFs with fibroblasts

Live neonatal fibroblast cells were cultured in media under standard conditions (37 °C, 5% CO_2, humidified incubator) to obtain good growth level. Each COF was dispersed in sterile PBS by mild sonication for 30 minutes to obtain uniform suspensions. Stock solutions were prepared and further diluted with culture medium to achieve final concentrations of (0, 1, 10, 50, and 150) µg/mL. The cell viability in response to COFs was assessed using the MTT assay. The fibroblast cells were seeded in 48-well plates at a density of 1×10^4 cells/well and incubated overnight to allow cell attachment. Cells were then treated with different concentrations of each COF separately for 48 hours. After treatment, 20 µL of MTT solution (5 mg/mL in PBS) was added to each well, and plates were incubated for 4 hours at 37 °C. The resulting formazan crystals were dissolved by adding 100 µL of dimethyl sulfoxide (DMSO) per well. Finally, the absorbance measurement was recorded at 540 nm using a microplate reader.

Electrochemical and statistical analysis

The double-layer capacitance (C_{dl}) values were extracted from the Nyquist plots by fitting the data to an equivalent circuit model using NOVA 12.1 software with a $\chi^2 > 0.09$. All statistical analyses were done using OriginPro Lab (2023b) software. The calibration curves for each

analyte tested represents the average C_{dl} values at a certain concentrations. The specific capacitance (Cs) values were calculated from the CV measurements at different scan rates using Eq. 1. The selectivity index (SI) calculation for each analyte was determined using Eq. 2. Linear and nonlinear regression analyses were performed to evaluate the goodness of fit for the experimental data.

$$Cs = \frac{\int idv}{[2mk(V_1 - V_2)]} \quad (1)$$

Where, Cs is the specific capacitance in F/g, $\int idv$ is the integrated area of the CV curve in AV, m is the mass of electroactive material of the electrode in g, k is the scan rate of CV in V/s, and V1-V2 is the scanned potential window of CV in V.

$$SI_{Analyte} = \frac{1}{N}\sum_{i=1}^{N}\left(\frac{\Delta Z_{Analyte}}{\Delta Z_{Glu}} \times 100\right) = \frac{1}{N}\sum_{i=1}^{N}\left(\frac{Z_{Analyte} - Z_{Analyte,Baseline}}{Z_{Glu} - Z_{Glu,Baseline}} \times 100\right) \quad (2)$$

Where, SI is the selectivity index, N is the number of concentrations measured, $Z_{Analyte}$ is the impedance response for a particular analyte, $Z_{Analyte,Baseline}$ is the impedance response for the electroactive electrolyte, Z_{Glu} is the impedance response for Glu, $Z_{Glu,Baseline}$ is the impedance response for the electroactive electrolyte.

The calibration curves were obtained by plotting the change in double layer capacitance as a function of glucose concentration over a broad range spanning from 1 aM to 5 mM. It is assumed that the instrument response y is linearly related to the log of standard glucose concentration across the tested concentration range (ax+b). The correlation coefficient (R^2) for all calibration curves exceeds 0.968, indicating excellent linearity. The limit of blank (LoB), limit of detection (LOD), and limit of quantification (LOQ) were estimated from the calibration equation based on the changes in the double-layer capacitance (Cdl), reflecting a high sensitivity to glucose as per the equations below,[2] and the repeatability of results were proved by successive measurements (n=3).

$$LoB = mean_{blank} + 1.645\ (SD_{blank}) \quad (3)$$

$$LOD = \frac{3\sigma}{S} \quad (4)$$

$$LOQ = \frac{10\sigma}{S} \quad (5)$$

Where, σ is the standard deviation of the blank solution, and S is the slope of the linear range of the calibration curve.

Stability Testing Simulated Physiological Conditions

The structural and functional stability of the COF-based sensors were evaluated in aqueous physiological environments, the electrode was immersed in PBS, pH 7.4, at 37 °C for 12, 24, 48 hours to simulate a physiological environment in terms of ionic strength, pH, and temperature. Following the immersion, the electrode was rinsed gently with deionized water and dried under ambient conditions before performing EIS and glucose sensing measurements to assess any degradation in the electrochemical properties or sensing performance. These results were compared with the freshly prepared COF-based electrode under identical conditions to determine retention of functionality.

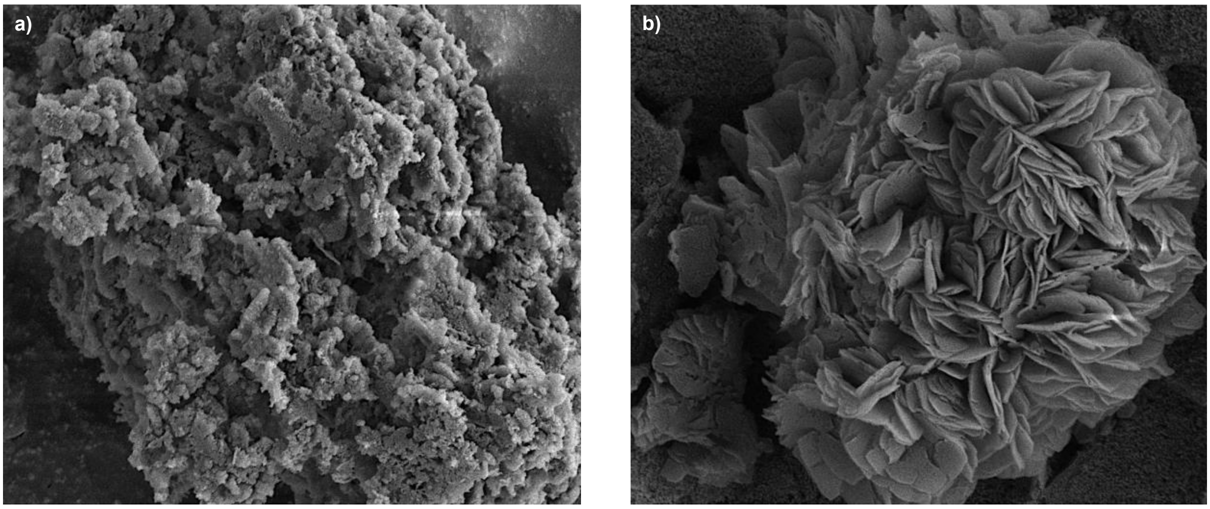

Figure S18. SEM images of a) TtaHz and b) TFPAHz after coating on SPCE electrodes.

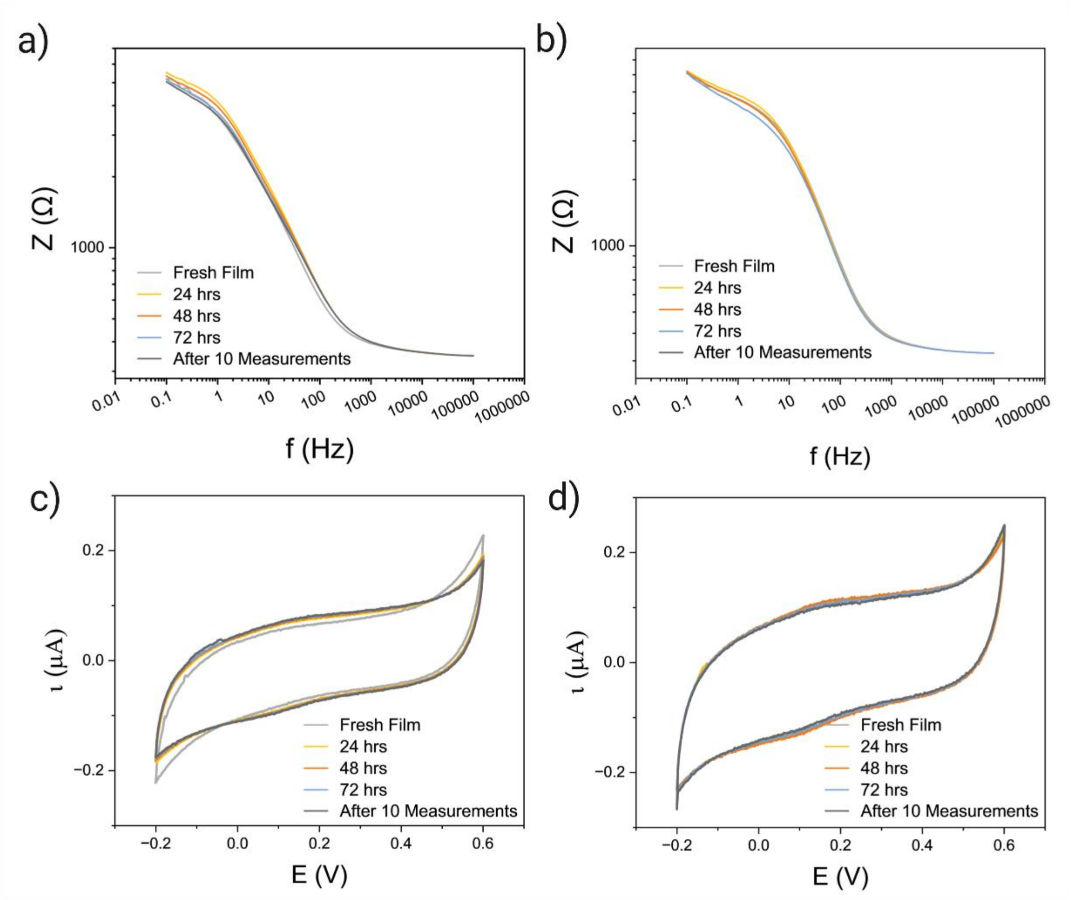

Figure S19. EIS bode plots highlighting the changes in impedance response of the coatings after 24, 48, and 72 hours, and after 10 electrochemical measurements for a) TtaHz and b) TFPAHz. CV curves of the coatings after the specified intervals, including after 10 measurements for c) TtaHz and d) TFPAHz.

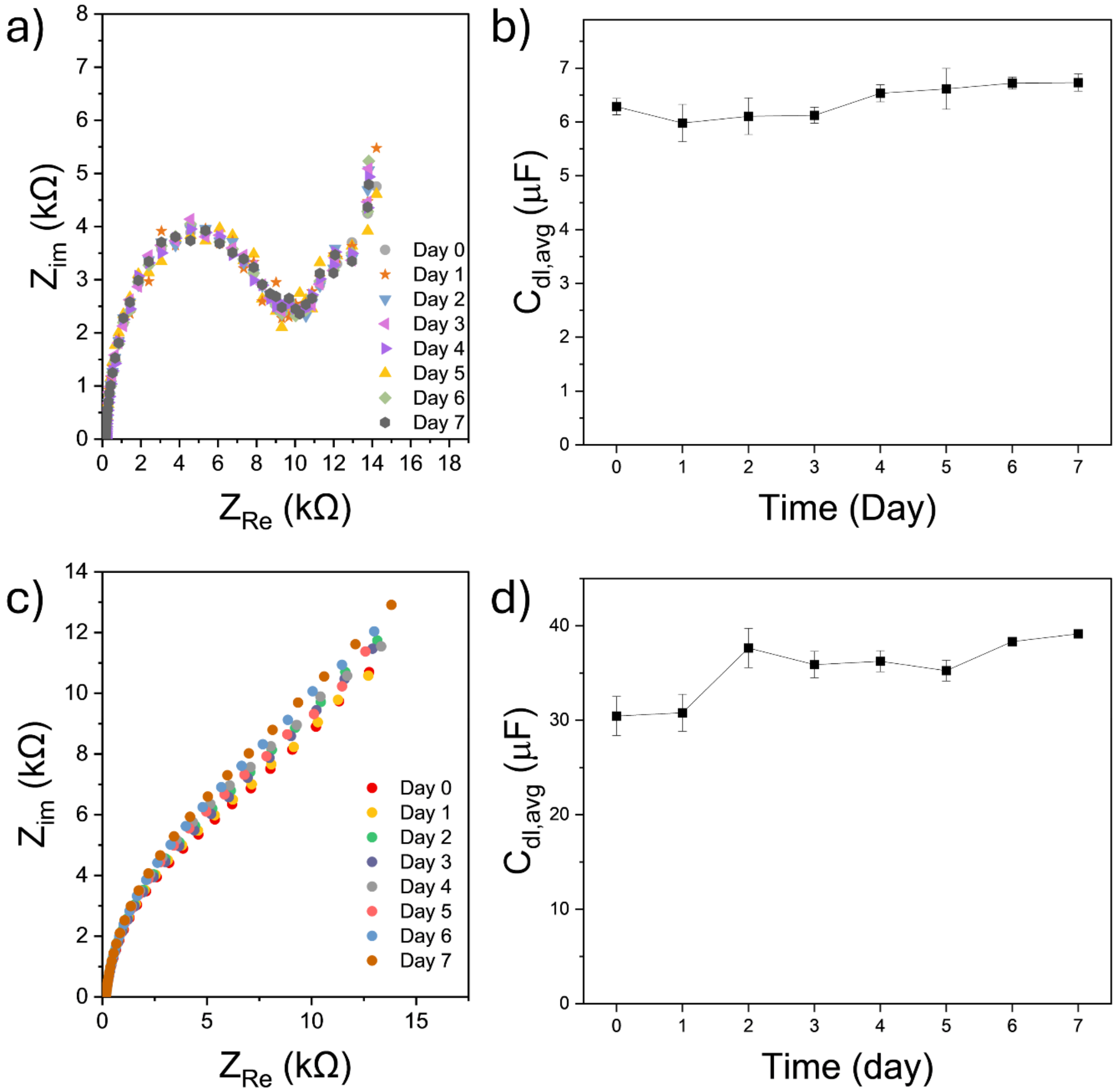

Figure S20. Long-term stability of the TFPAHz-coated electrode over 7 days. a) Nyquist plots in 5 mM $K_3[Fe(CN)_6]$ over 7 days. b) Corresponding calibration curve of average (Cdl) over 7 days. c) Nyquist plots of the response in 1 nM glucose, demonstrating sensing response over 7 days. d) Corresponding calibration curve showing glucose response over 7 days.

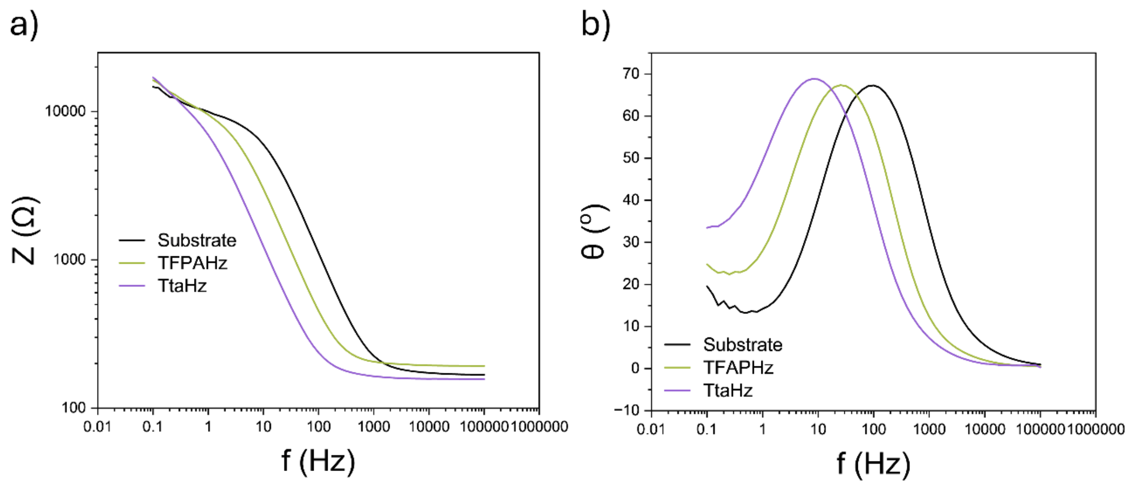

Figure S21. EIS of SPCE, TFPAHz-coated SPCE, and TtaHz-coated SPCE. a) Bode plots highlighting the electrochemical response and charge transport behavior of each electrode configuration. b) Phase angle plots indicating the capacitive and resistive contributions across the frequency range.

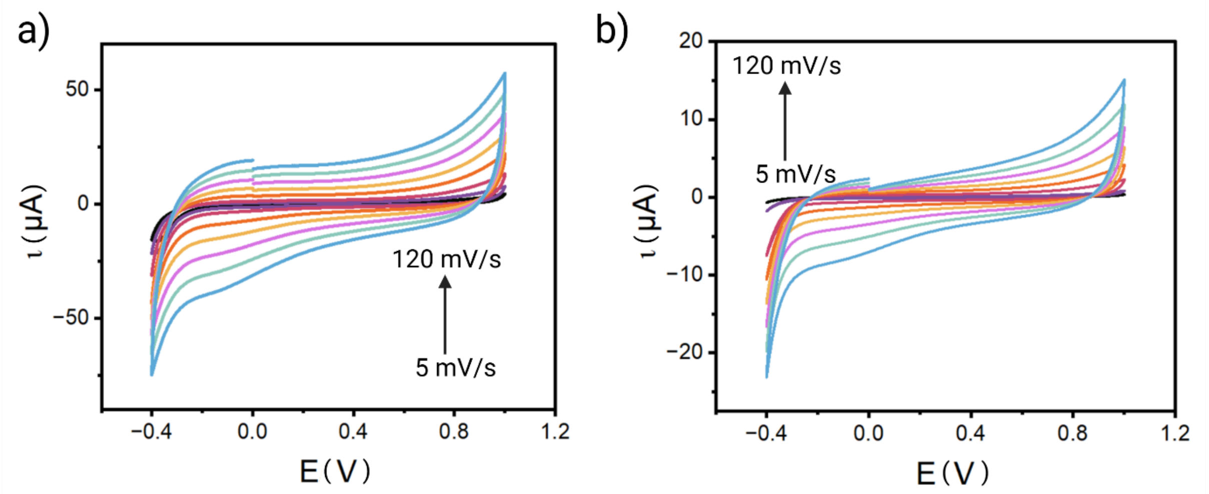

Figure S22. a) CV of TFPAHz-coated electrode in DPBS (×1) vs. Ag/AgCl at varying scan rates (5, 10, 20, 40, 50, 80, 100, and 120 mV/s). b) CV of TtaHz-coated electrode in DPBS (×1) vs. Ag/AgCl at same scan rates.

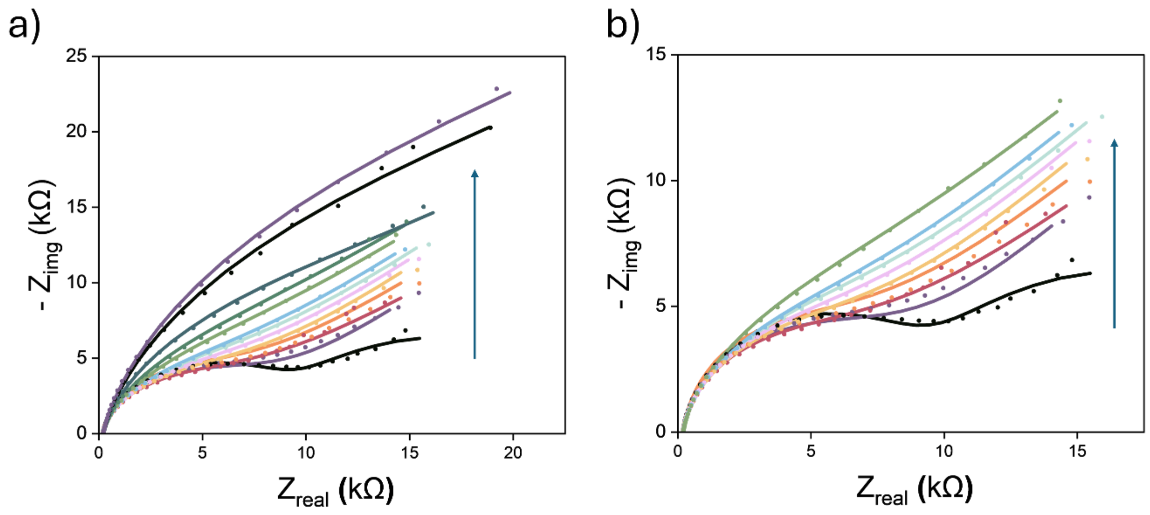

Figure S23. Overlay of the experimental data of TFPAHz-coated electrode and the simulated Nyquist plots fitted using the equivalent circuit in response to increasing glucose concentration vs. Ag/AgCl in 5 mM $K_3[Fe(CN)_6]$ for a) Full concentration range (1 aM – 5 mM) and b) aM–nM range.

.

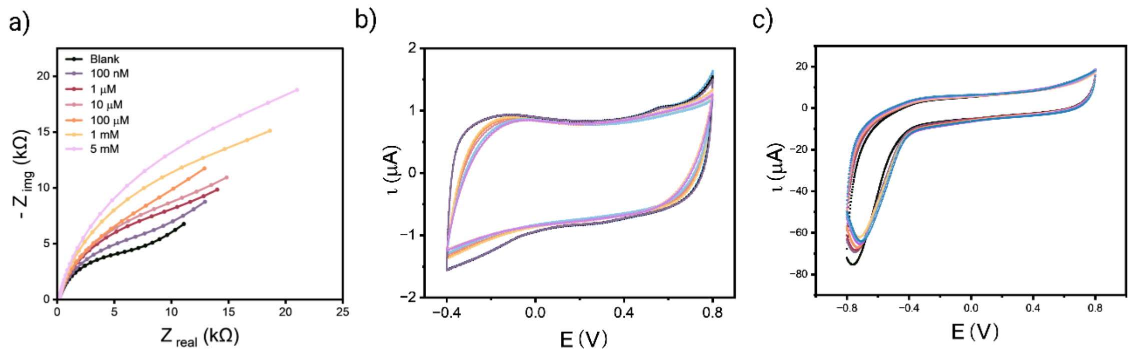

Figure S24. a) Overlay of the experimental data of TtaHz-coated electrode and the simulated Nyquist plots fitted using the equivalent circuit in response to increasing glucose concentration vs. Ag/AgCl in 5 mM $K_3[Fe(CN)_6]$. CV curves of glucose sensing at the COF-modified electrode recorded at concentration values of [0 M, 100 aM, 100 fM, 100 pM, 100 nM, 100 µM, 1mM, 5 mM] in DPBS for b) TFPAHz c) TtaHz.

Table S2. The linearity and sensitivity summary of glucose sensing using both COFs.

COF Structure	Regression equation $(ax+b)$	R^2	Linear Range	LOD	LOQ	LoB (µF)
TFPAHz	$(1.93 \bullet 10^{-6})x + (2.32 \bullet 10^{-5})$	0.968	1 aM – 5 mM	0.878 aM	2.66 aM	6.69×10^{-6}
TtaHz	$(1.84 \bullet 10^{-6})x + (1.95 \bullet 10^{-5})$	0.994	100 nM – 5 mM	4.87 nM	14.75 nM	7.47×10^{-6}

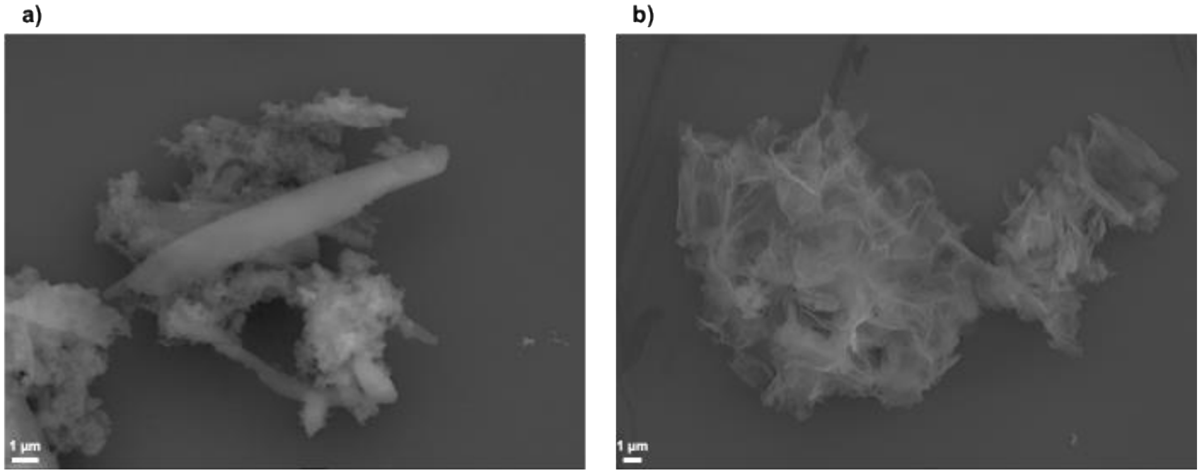

Figure S25. SEM images of a) TtaHz and b) TFPAHz after the sensing process.

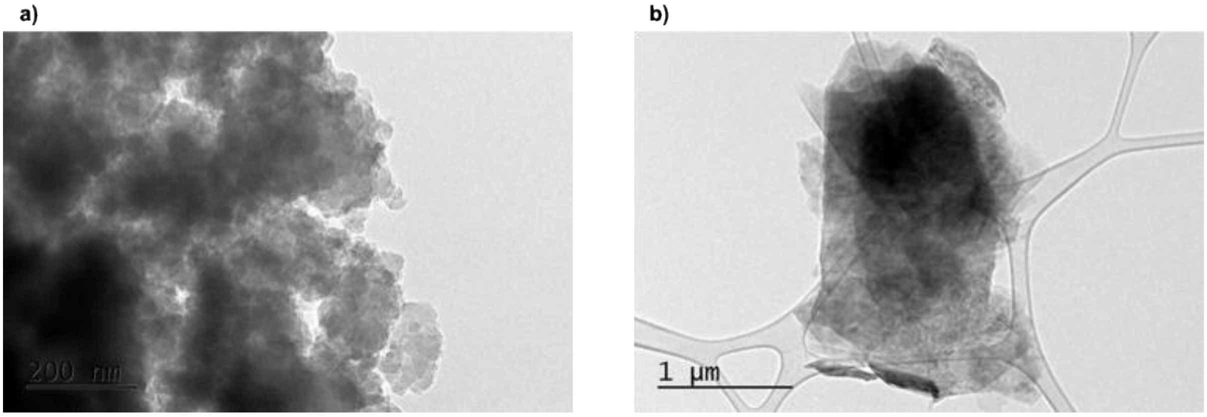

Figure S26. TEM images of a) TtaHz and b) TFPAHz after the sensing process.

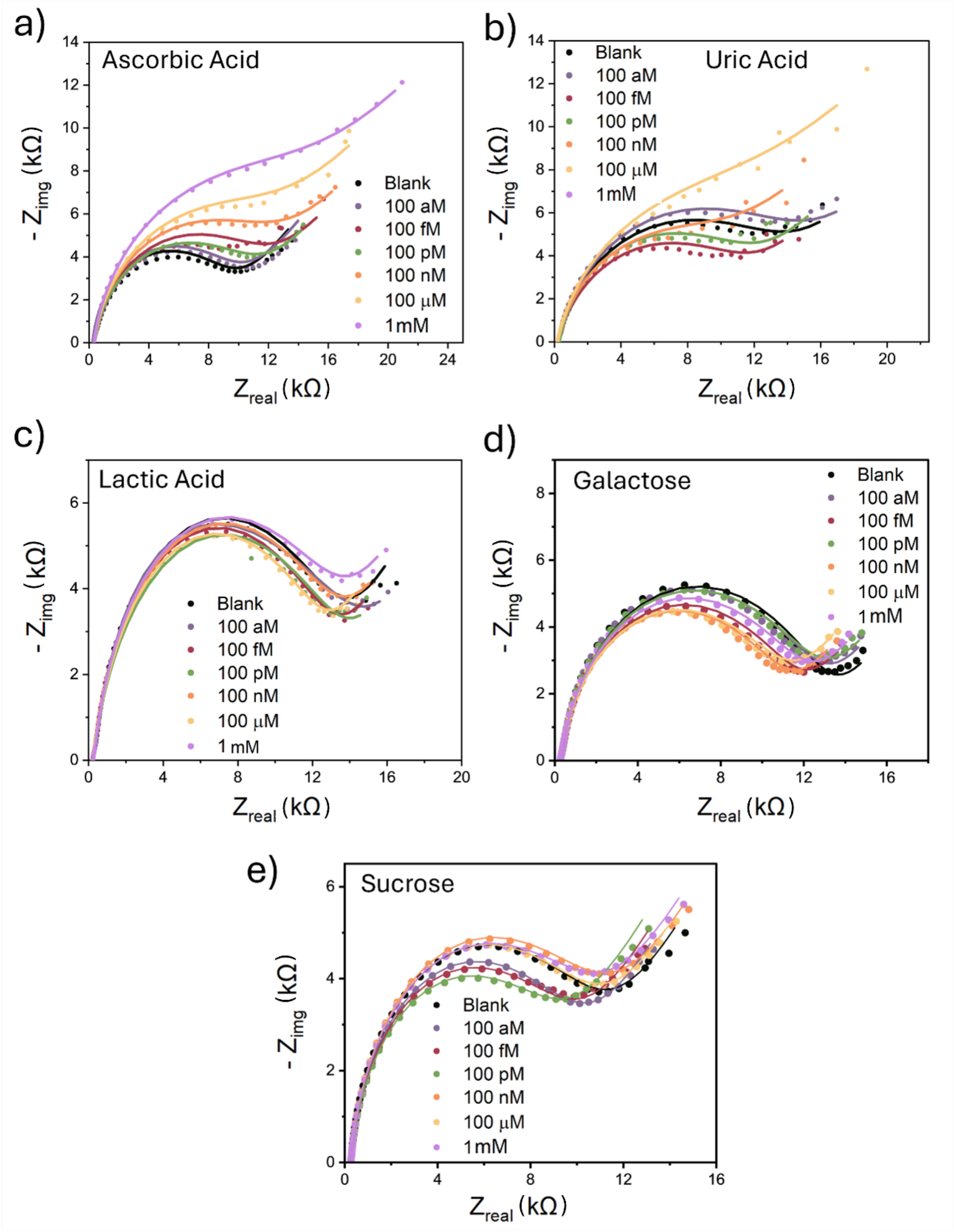

Figure S27. Nyquist plots showing the electrochemical response of the TFPAHz-coated electrode to various analytes each tested at concentrations of 0 M, 100 aM, 100 fM, 100 pM, 100 nM, 100 uM, 1 mM.

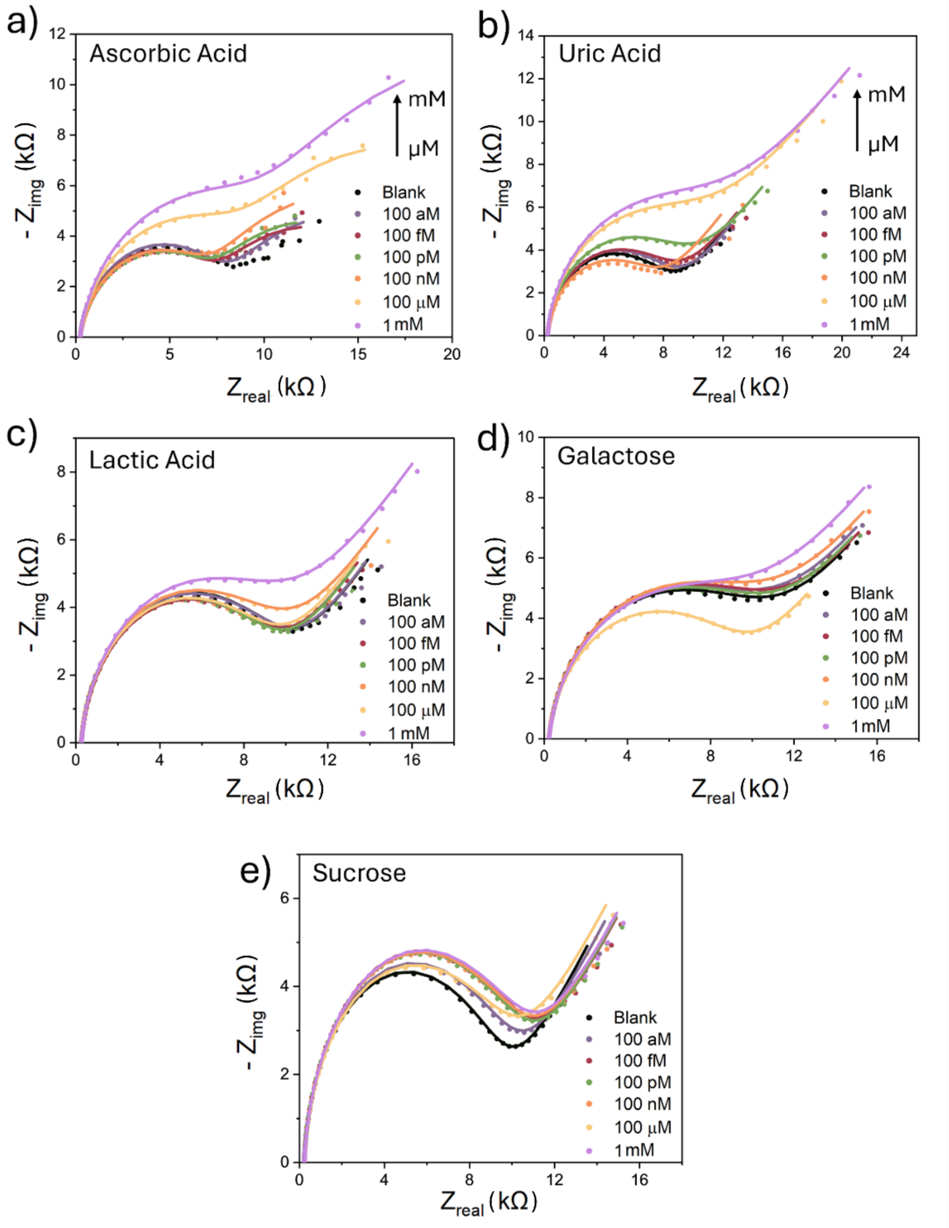

Figure S28. Nyquist plots showing the electrochemical response of the TtaHz-coated electrode to various analytes each tested at concentrations of 0 M, 100 aM, 100 fM, 100 pM, 100 nM, 100 uM, 1 mM.

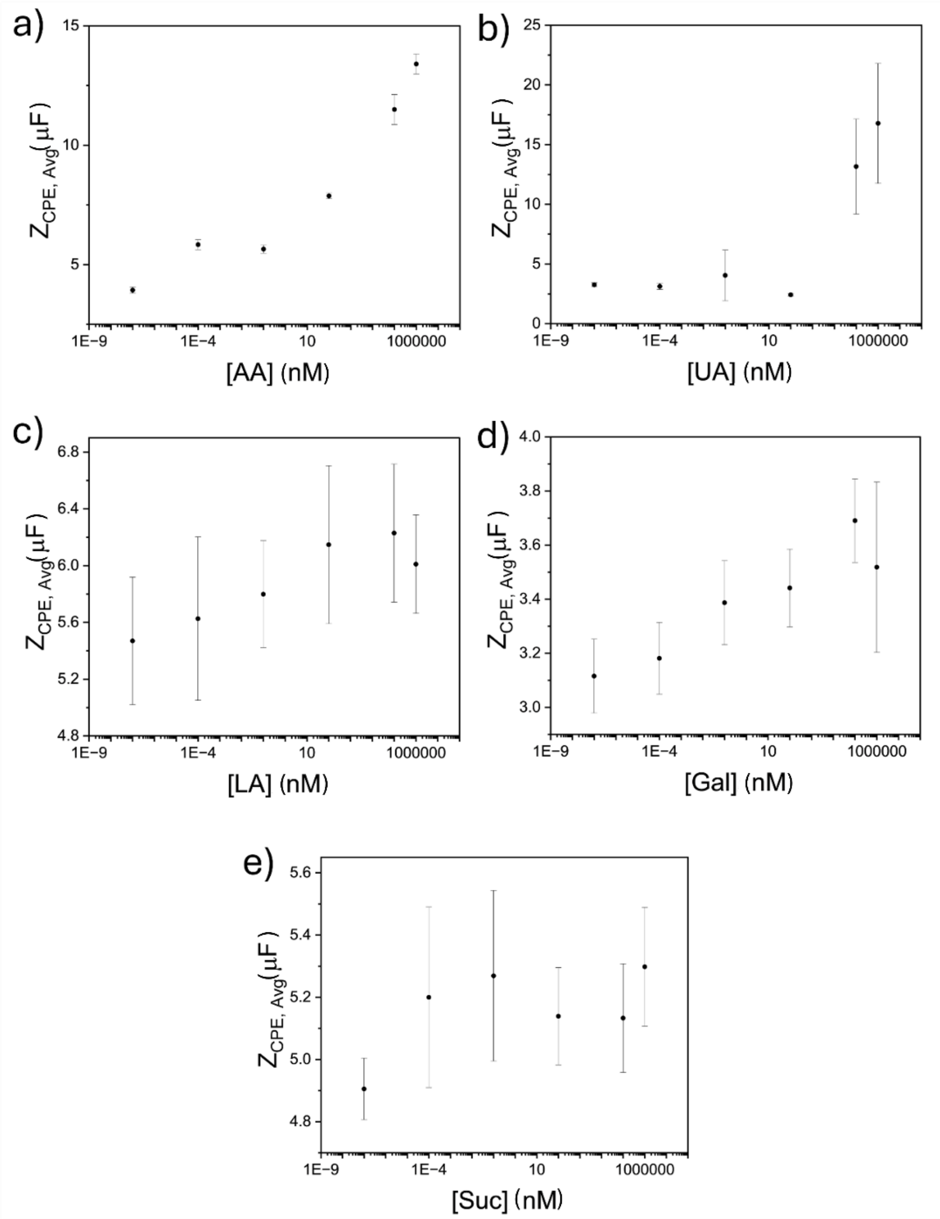

Figure S29. Selectivity results for TFPAHz showing the calibration curve of the Z_{CPE} values as a function of analyte concentration, plotted on a semilogarithmic scale.

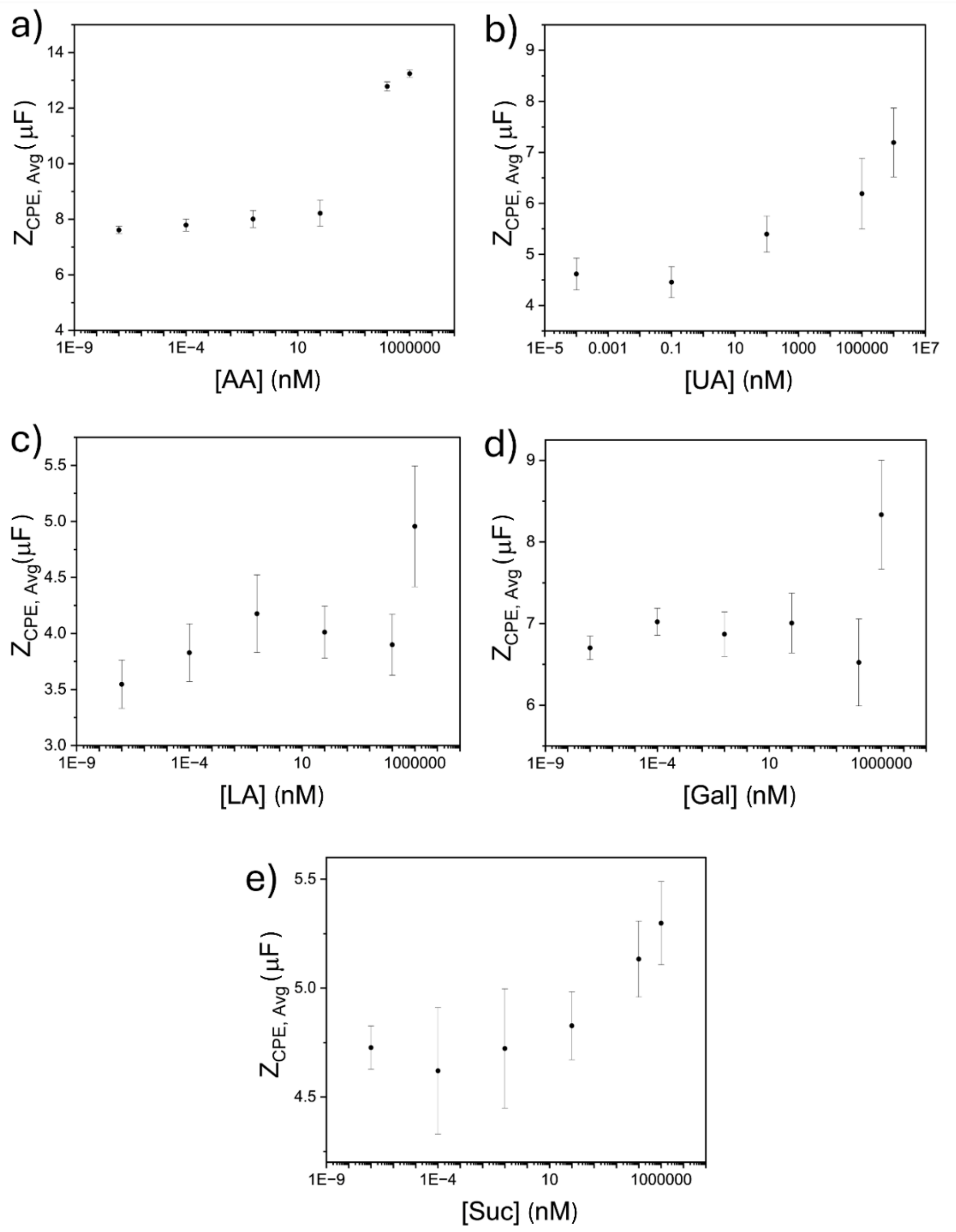

Figure S30. Selectivity results for TtaHz showing the calibration curve of the Z_{CPE} values as a function of analyte concentration, plotted on a semilogarithmic scale.

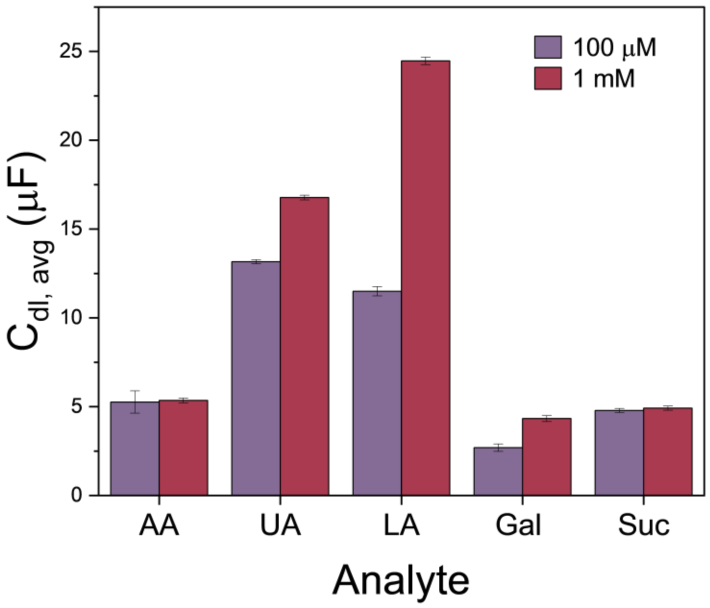

Figure S31. Average Z_{CPE} values for all analytes tested on TFPAHz-coated electrode and are measured at concentrations of 100 μM and 1 mM. Data represent the mean ± standard deviation from three independent measurements.

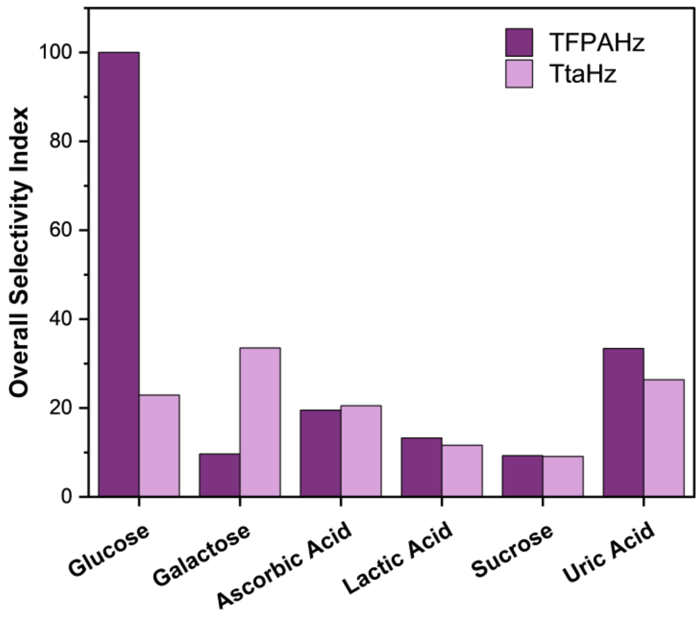

Figure S32. Overall selectivity index for all the analytes tested on TFPAHz and TtaHz.

Table S3. Statistical significance summary of the TFPAHz-coated electrode response to different tested analytes representing P-values of the t-test analysis performed between pairs of different analytes (P-value < 0.05: significant difference; P-values > 0.05: no significant difference).

Pair-Sample t-Test	P-value
Glu-LA	0.00228
Glu-Gal	0.0027
Glu - Suc	0.003
Glu – UA	0.00265
Glu-AA	0.0046
AA-UA	5.82406E-4
AA-LA	0.02714
AA-Gal	0.00113
AA-Suc	0.00299
UA-LA	0.00719
UA-Gal	0.0041
UA-Suc	3.36814E-4
LA-Gal	0.01256
LA-Suc	0.1286
Gal-Suc	2.2659E-4
Interferent – interferent with Glu	<0.0001
Glu – Blank ($K_3[Fe(CN)_6]$)	0.04131
FBS – Blank ($K_3[Fe(CN)_6]$)	3.35004E-4
Glu - FBS	0.31451

Table S4. Analytical performance of non-enzymatic electrochemical glucose sensors.

Sensing system	Detection method	Linear range	LOD	Reference
Au@CuO/LIG	Amperometric	0.005 mM - 5 mM	1.8 µM	[3]
3D-KSC/$COF_{TAPB-PDA}$/CuNPs	Amperometric	4.69 µM - 1.57 mM and 1.57 mM - 7.07 mM	1.54 µM	[4]
HS-260	Amperometric	0.5 µM -965.5 µM	3.9 µM	[5]
Ni_2P/G	Amperometric	5 µM - 1.4 mM	0.44 µM	[6]
Ni/NiO/NC	Amperometric	0.6 µM -8.6 mM	0.2 µM	[7]
Ni@C/Ni	Amperometric	0.15 µM - 1.48 mM	50 nM	[8]
1-CF	Amperometric	2 µM - 2 mM	0.1 µM	[9]
PNMOF	Amperometric	0.5 µm - 2665.5 µm	-	[10]
Cu-MHOF	Amperometric	0.1 µM - 22 mM	86 nM	[11]
CuONPs/Ce-MOF	Amperometric	5 nM - 8.6 mM	2 nM	[12]
MIP@Ni foam	Impedimetric	10 mM - 55 mM	-	[13]
PoPD/Ag hybrid-modified GCE	Impedimetric	1.14 mM to 2.66 mM	1.78 µM	[14]
Cu,N-CQDs	Impedimetric	5 µM - 700 µM	1.22 µM	[15]
NiO/GCE	Impedimetric	5 µM - 1 mM and 1 mM - 6 mM	1.1 µM	[16]
**TtaHz**	**Impedimetric**	**100 nM - 5 mM**	**4.87 nM**	**This work**
**TFPAHz**	**Impedimetric**	**1 aM - 5 mM**	**0.878 aM**	

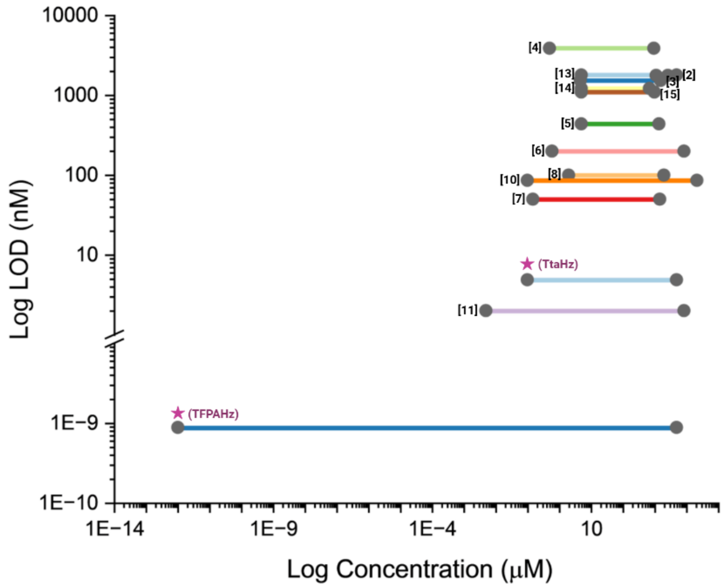

Figure S33. Comparative performance of recent non-enzymatic glucose sensors. The plot summarizes key metrics including linear detection range and LOD of previous electrochemical glucose sensors comparing with the proposed azine-linked COF sensor (highlighted with a star), demonstrating superior attomolar sensitivity and wide detection range for TFPAHz. Data were extracted from 14 peer-reviewed studies published between [2017–2025].

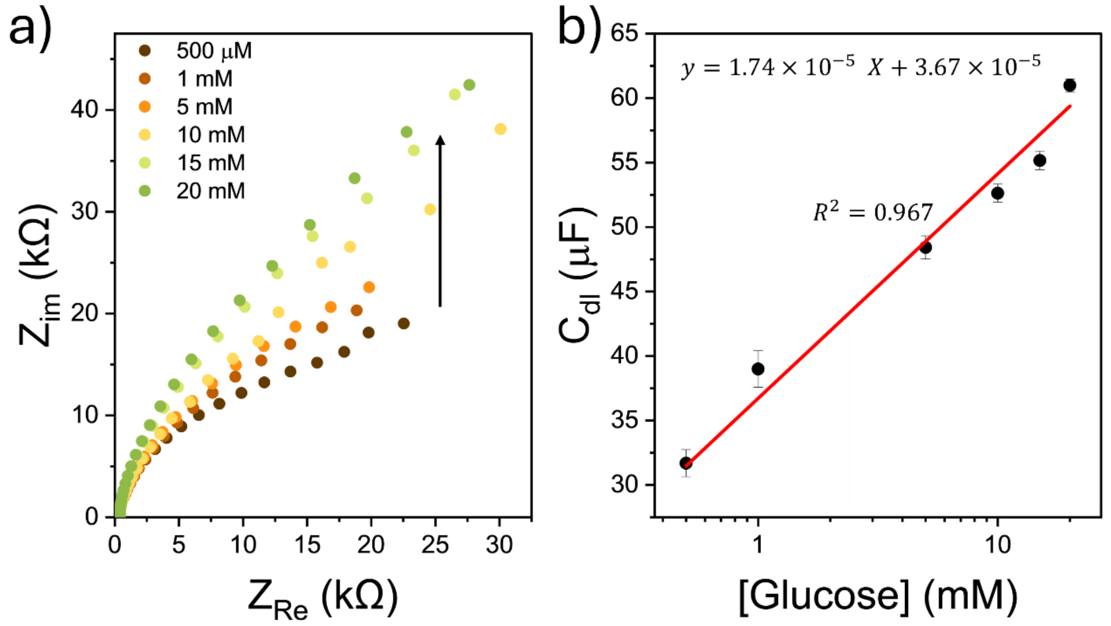

Figure S34. EIS response for high glucose concentration sensing on TFPAHz-coated electrode with calibration curves. (a) Nyquist plots of the TFPAHz-coated electrode recorded in 5 mM probe solution at glucose concentrations (0.5–20 mM), showing systematic changes in the impedance spectra with increasing glucose concentration. (b) Double-layer capacitance (Cdl) extracted from EIS fitting vs. glucose concentration.

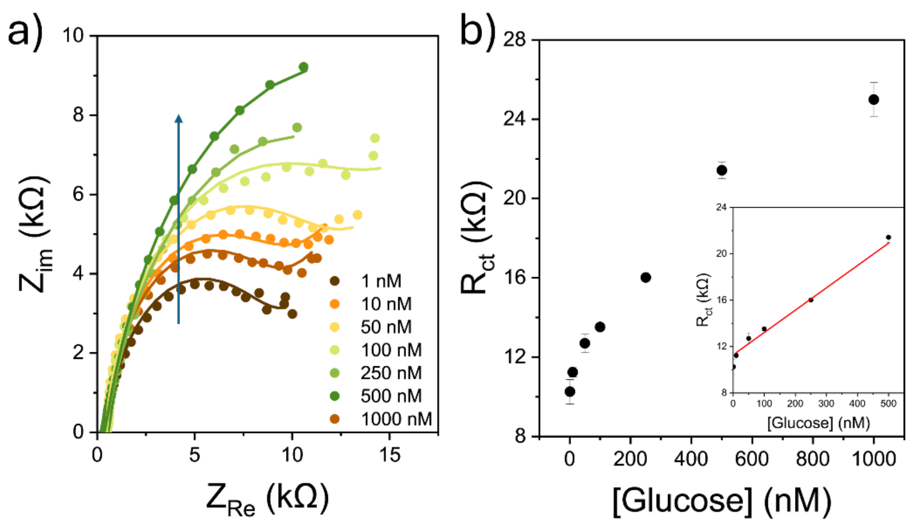

Figure S35. Electrochemical response of the sensor in artificial sweat samples. a) Overlay of experimental Nyquist plots of the TFPAHz-coated electrode in artificial sweat samples spiked with glucose (1–1000 nM) and the corresponding simulated plots obtained from fitting with the equivalent circuit, recorded versus Ag/AgCl reference electrode. b) Corresponding calibration curve derived from the charge-transfer resistance values as a function of glucose concentration with inset for the linear range (10 – 500) nM.

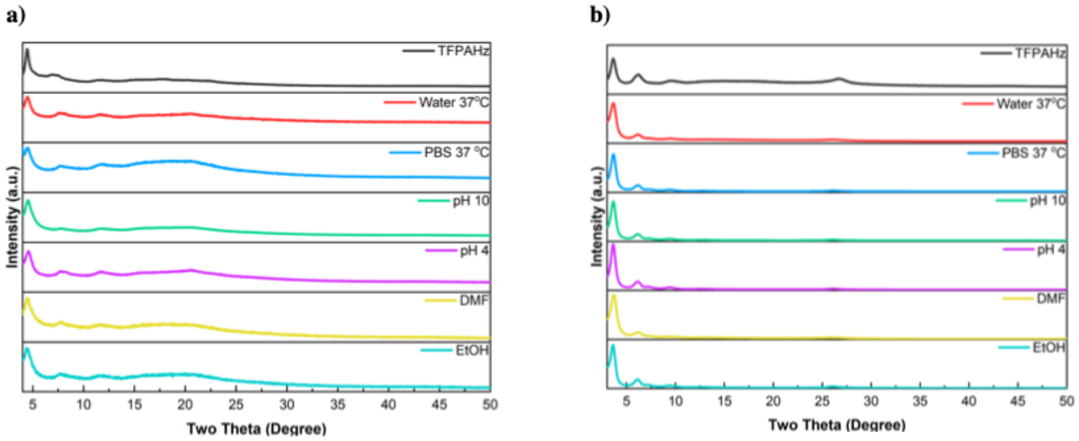

Figure S36. PXRD profile of a) TFPAHz and b) TtaHz after incubation in different solvents for 7 days.

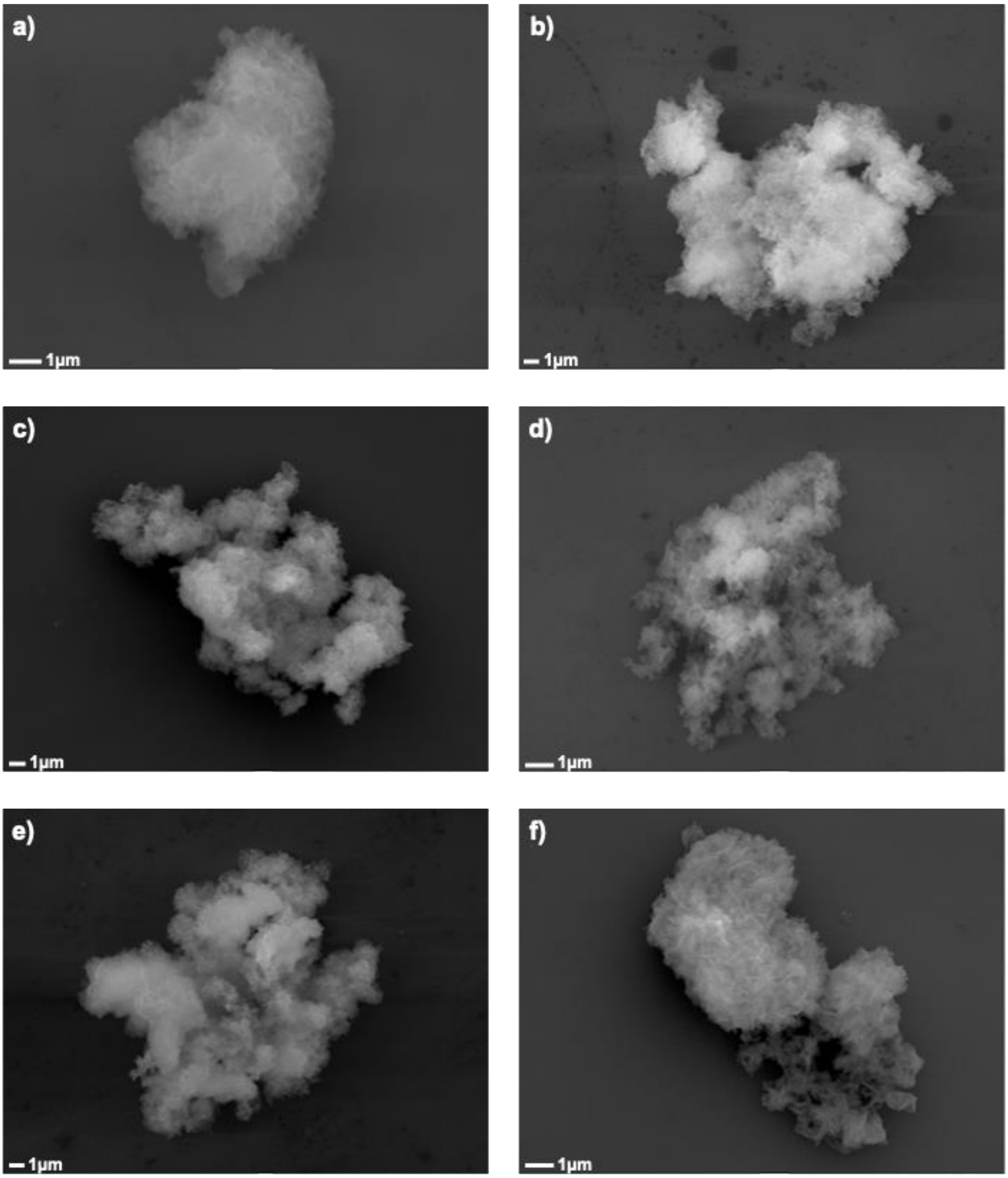

Figure S37. SEM images of TFPAHz after incubation for 7 days in a) DMF, b) EtOH, c) pH4, d) pH 10, e) Water at 37 °C, and f) PBS at 37 °C.

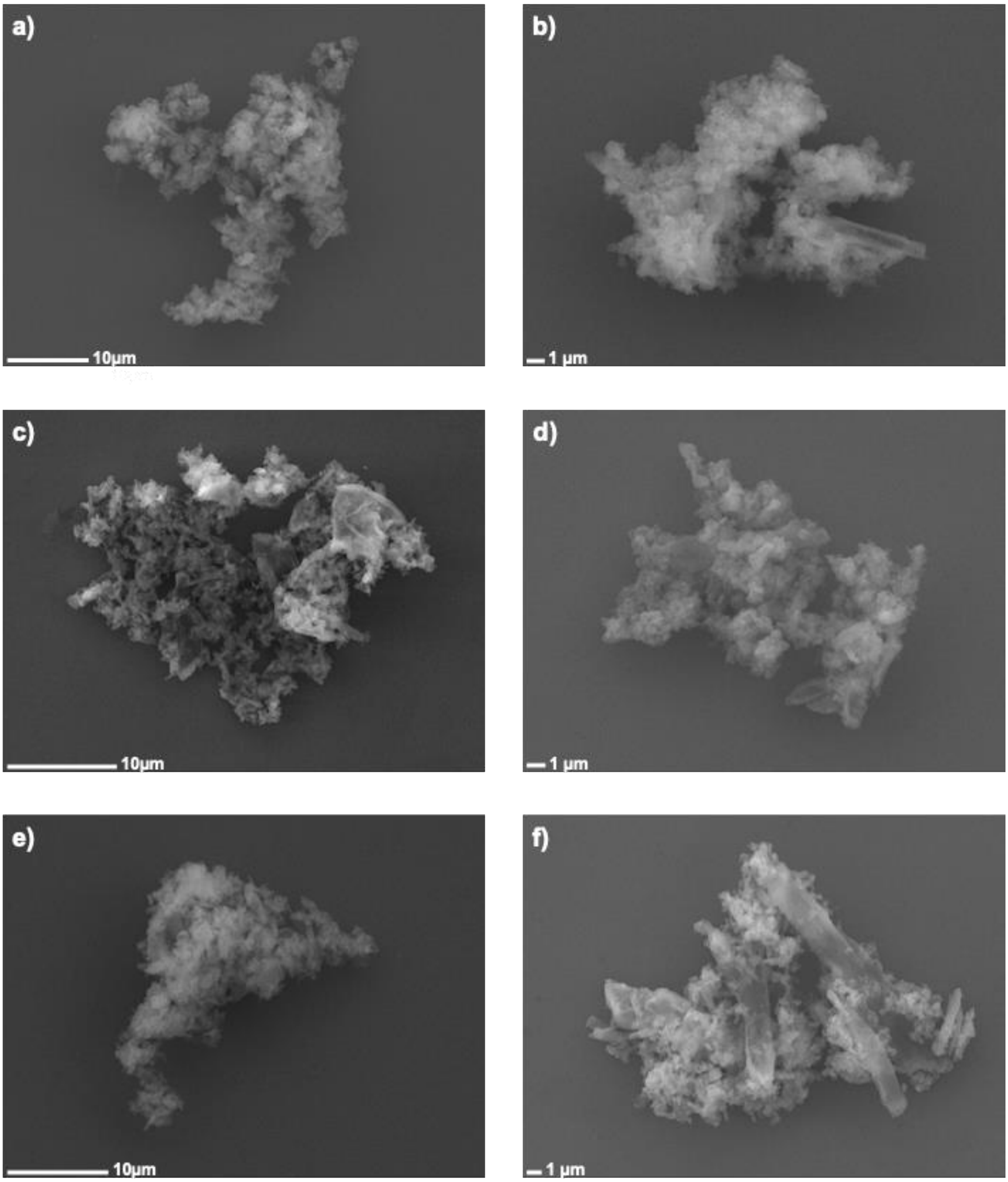

Figure S38. SEM images of TtaHz after incubation for 7 days in a) DMF, b) EtOH, c) pH4, d) pH 10, e) Water at 37 °C, and f) PBS at 37 °C.

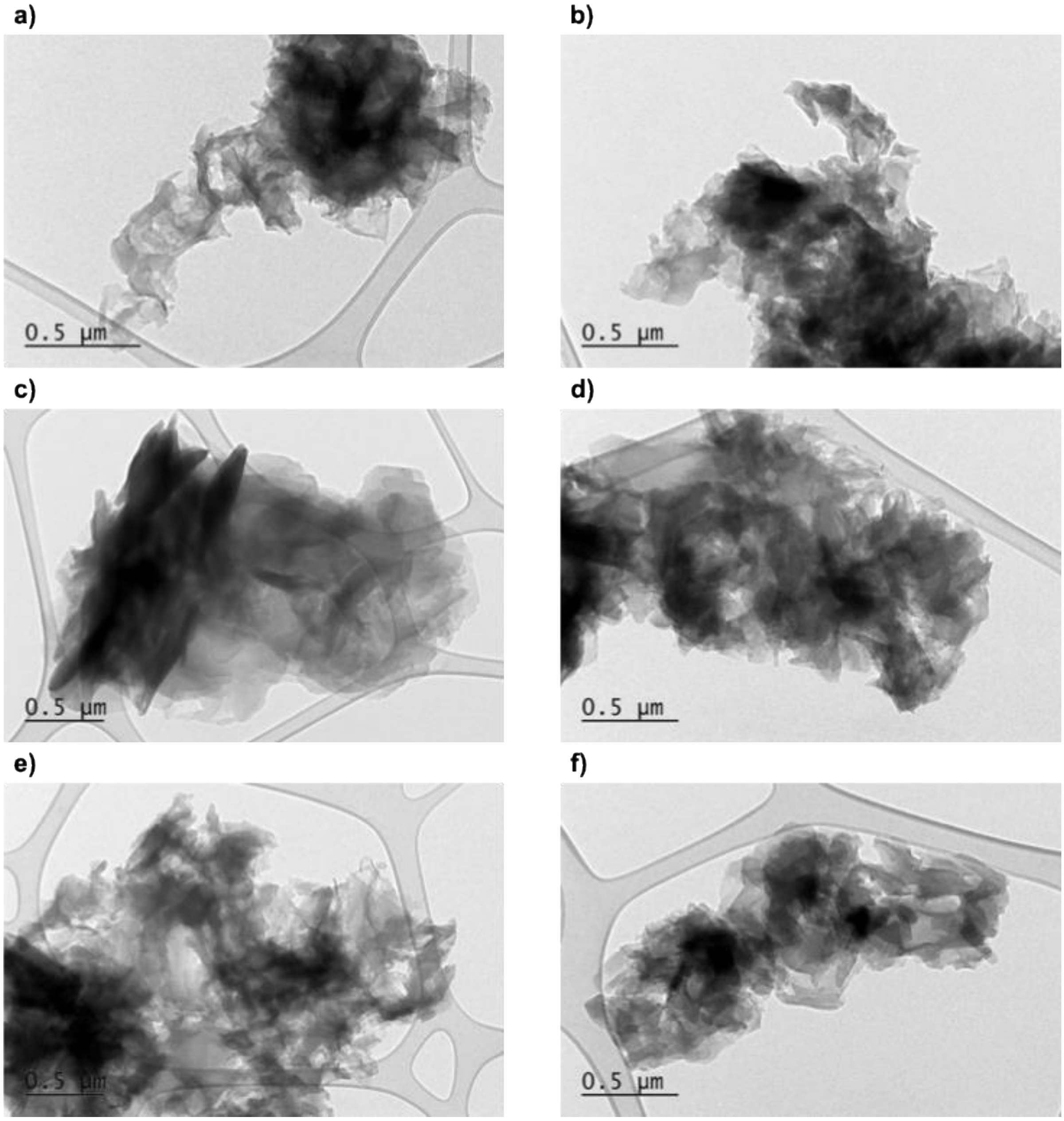

Figure S39. TEM images of TFPAHz after incubation for 7 days in a) DMF, b) EtOH, c) pH4, d) pH 10, e) Water at 37 °C, and f) PBS at 37 °C.

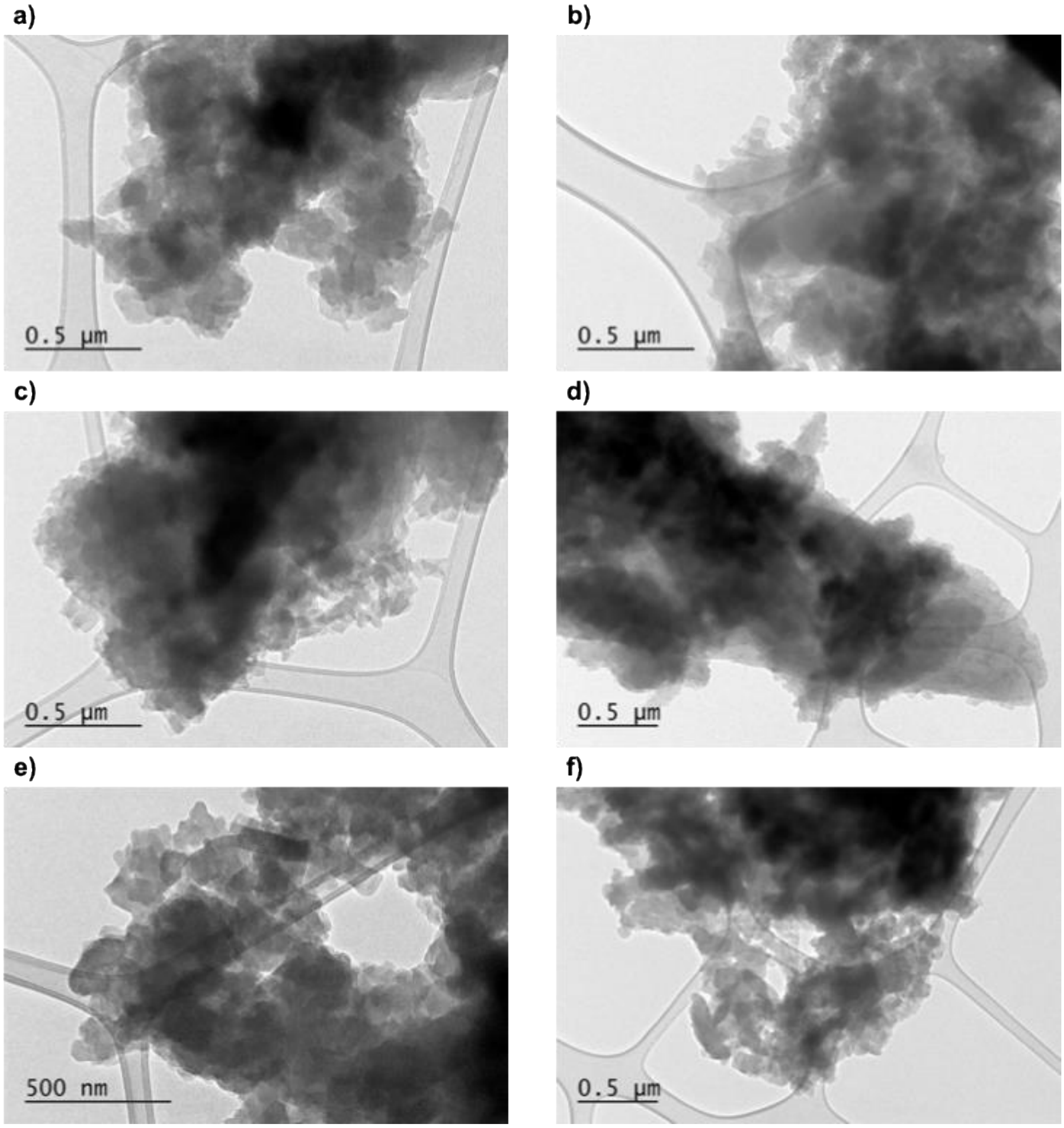

Figure S40. TEM images of TtaHz after incubation for 7 days in a) DMF, b) EtOH, c) pH4, d) pH 10, e) Water at 37 °C, and f) PBS at 37 °C.

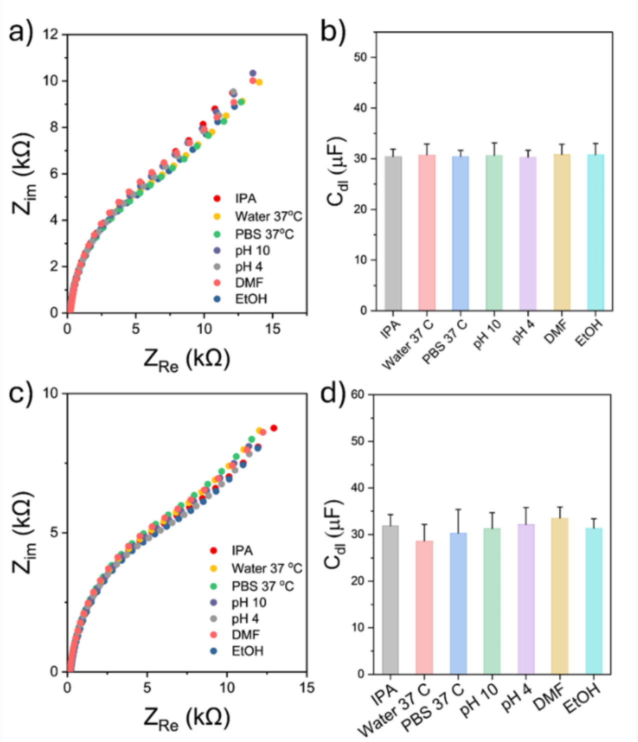

Figure S41. Evaluation of functional stability in various polar and physiologically relevant media. a) Nyquist plots of TFPAHz-coated electrodes after incubating in different solvents for 7 days measured at 1 nM glucose (IPA, water (37 °C), PBS (37 °C), pH 4, pH 10, DMF, and EtOH). b) Corresponding bar graph of the average C_{dl} values (n = 3). c) Nyquist plots of TtaHz-coated electrodes after incubating in different solvents measured at 100 nM glucose. d) Corresponding bar graph of average C_{dl} values (n = 3).

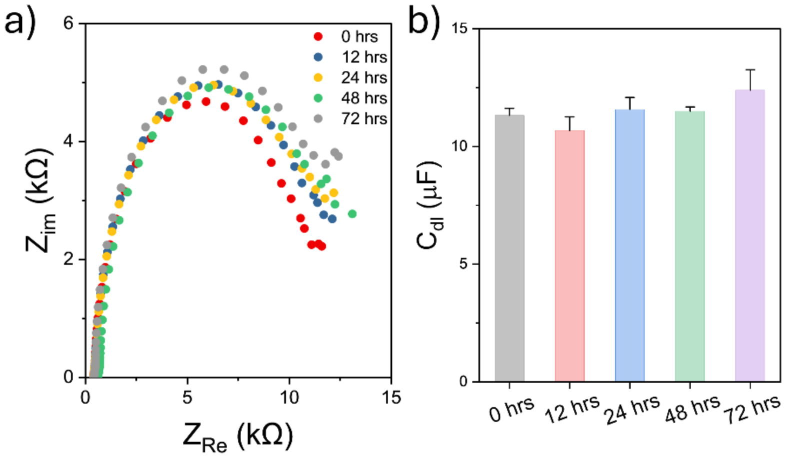

Figure S42. Assessment of glucose sensing performance of TFPAHz-coated electrodes over time in a physiologically-relevant environment. a) Nyquist plots of TFPAHz-coated electrodes immersed in FBS at 37 °C for 0, 12, 24, 48, and 72 h, measured at 10 nM glucose. b) Bar graph of corresponding average C_{dl} values at each time point.

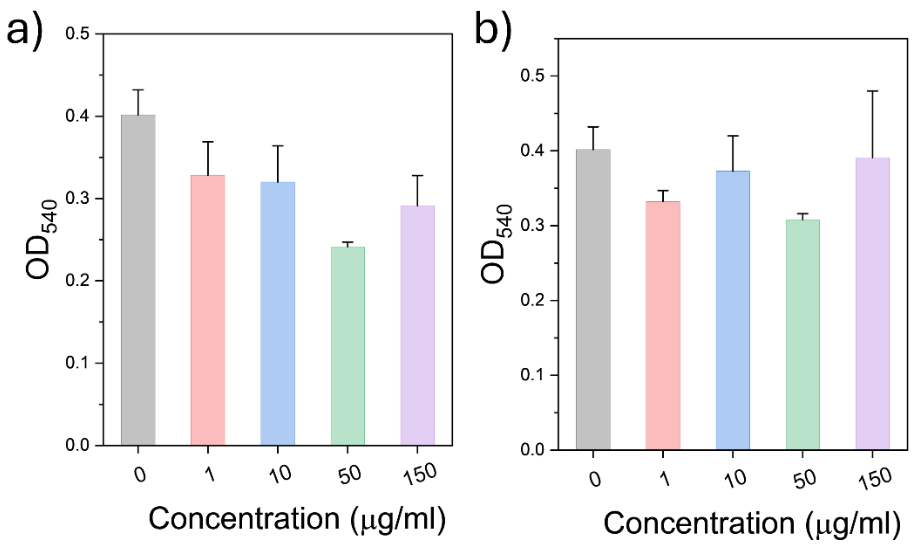

Figure S43. Cell viability of fibroblast cells after incubation with different concentrations of COFs (0, 1, 10, 50, and 150 μg/mL). a) Viability profile for TFPAHz, b) Viability profile for TtaHz. Data are presented as mean ± SD (n = 3).

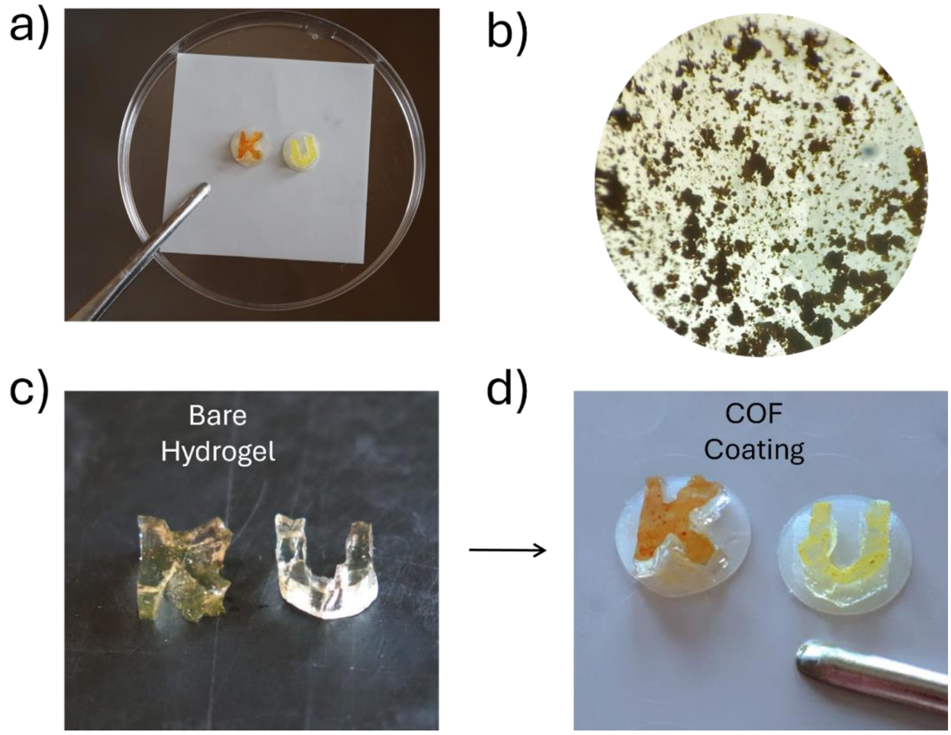

Figure S44. Hydrogel-based COF sensor platform for wearable glucose monitoring. a) hydrogel substrate coated with COF materials, TFPAHz to the left and TtAHz to the right. b) Microscope image showing surface coverage of the COF coating on the hydrogel surface. The hydrogel c) before and d) after COF coating.

S-12: Theoretical Calculations

Theoretical calculations

A comprehensive series of Density Functional Theory (DFT)-based calculations was conducted to explore the structural and electronic properties of TFPAHz and TtaHz, with a particular focus on their capabilities to stabilize D-glucose. The initial theoretical study focused on pre-optimization of the molecular building blocks constituting the COFs and the D-glucose molecule utilizing DFT as implemented in the Gaussian16 package at the M06-2X/6-311++G(d,p) level of theory.[17–19] The geometries of the D-glucose molecule and the molecular building blocks were systematically optimized, allowing full flexibility in all degrees of freedom, and their equilibrium configurations were verified through frequency calculations.

Building upon the pre-optimized molecular fragments, periodic boundary conditions were employed to model the extended 2D layered network structures of TFPAHz and TtaHz. Simultaneous structure and cell optimizations were carried out for a series of stacked 3D layered configurations derived from the canonical 2D structures. The construction of the 2D networks was performed using the QUANTUM ESPRESSO plane-wave DFT code,[20] with the GGA-PBESol functional applied to account for exchange-correlation (XC) effects.[21] To incorporate dispersion interactions, the Grimme DFT-D3 semi-empirical van der Waals correction was implemented.[22] Ultra-soft pseudopotentials were used to model ion-electron interactions within atomic species.[23] The Brillouin zones were sampled using optimized Monkhorst-Pack grids of [2×2×1] for 2D layers and [2×2×8] for 3D crystals.[24] One-electron wave functions were expanded on a plane-wave basis with kinetic energy cutoffs of 40 Ry for wave functions and 300 Ry for electronic density, ensuring accurate convergence of total energy and electronic density. For 3D crystal models, atomic relaxations were performed using a conjugate gradient minimization algorithm until the maximum force on any atom was reduced below 0.02 eV $Å^{-1}$, allowing for relaxation of interlayer distances. To enhance the treatment of the electronic correlation in the different systems considered, a Hubbard-corrected DFT+U approach was employed,[25] considering an optimized Hubbard parameter of U=4 eV.[26]

Minimum energy paths and activation barriers for the on-surface D-glucose diffusion on TFPAHz surface from an equilibrium starting position to an equivalent neighbor position have been calculated using the Climbing-Image Nudged Elastic Band (CI-NEB) approach.[27] Within the CI-NEB protocol, the initial, final, and sufficient number of intermediate image-states (20 in the present case) for the diffusion path were free to fully relax.

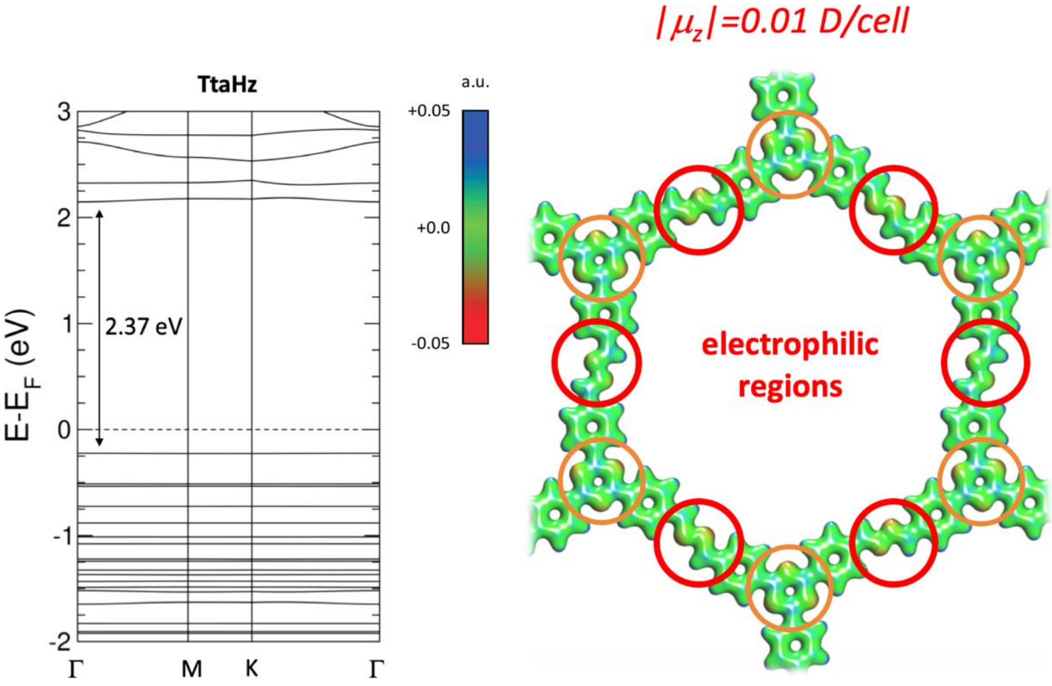

Figure S45. (left) DFT+U computed band structure for TtaHz crystal bulk (band gap of 2.37 eV at Γ point is indicated superimposed). (right) Electrostatic potential 3D colored-map isosurface for the TtaHz layer, where the most electrophilic regions are indicated by red circles, as well as the out-of-plane almost null dipole moment value of 0.01 Debyes per unit cell.

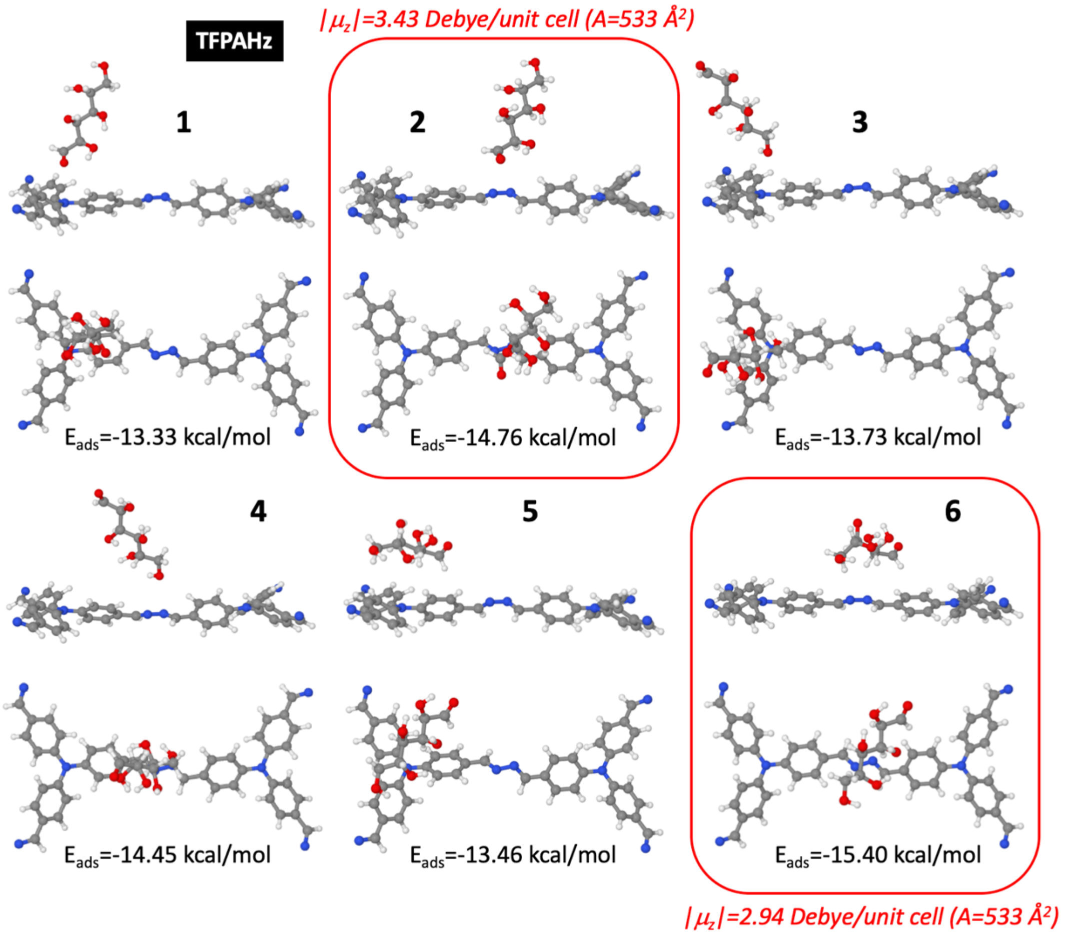

Figure S46. Six DFT-optimized most stable adsorption configurations found for D-glucose in interaction with the TFPAHz surface, with molecular adsorption energies ranging between -15.40 and -13.33 kcal/mol. Two most stable adsorption configurations have been highlighted, indicating their out-of-plane dipole moments of 2.94 and 3.43 Debyes per unit cell, respectively.

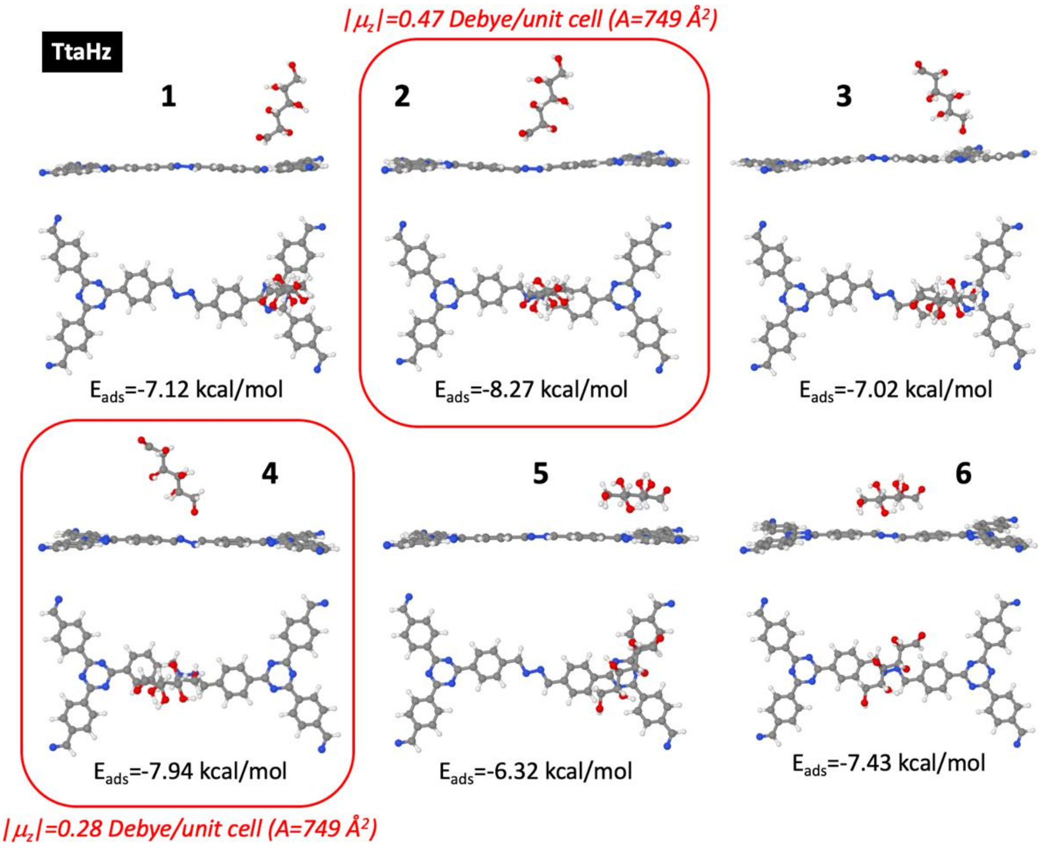

Figure S47. Six DFT-optimized most stable adsorption configurations found for D-glucose in interaction with the TtaHz surface, with molecular adsorption energies ranging between -8.27 and -6.32 kcal/mol. Two most stable adsorption configurations have been highlighted, indicating their out-of-plane dipole moments of 0.47 and 0.28 Debyes per unit cell, respectively.

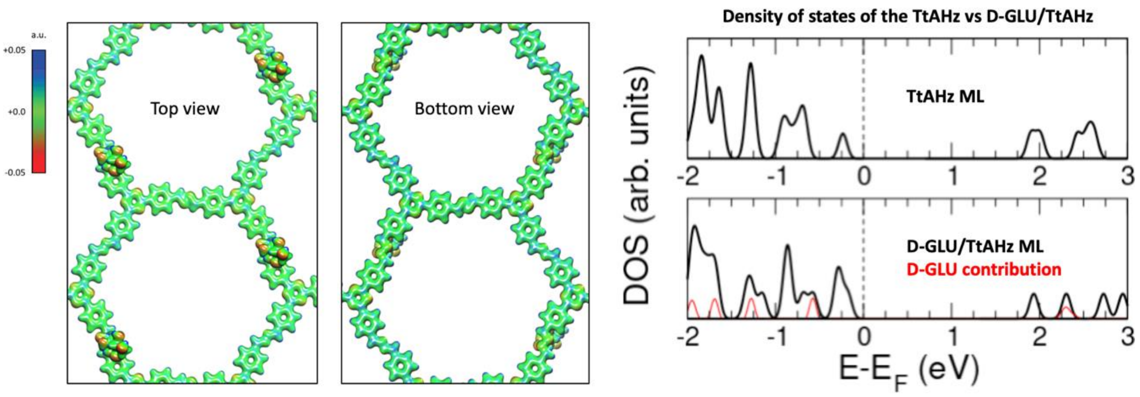

Figure S48. (left) Top and bottom views of the electrostatic potential 3D colored-map isosurface for the most stable D-glucose/TtaHz adsorption configuration. (right) Comparison between the density of states profiles of the pristine TtaHz monolayer vs the most stable D-glucose/TtaHz adsorption configuration, indicating in the later the molecular contribution.

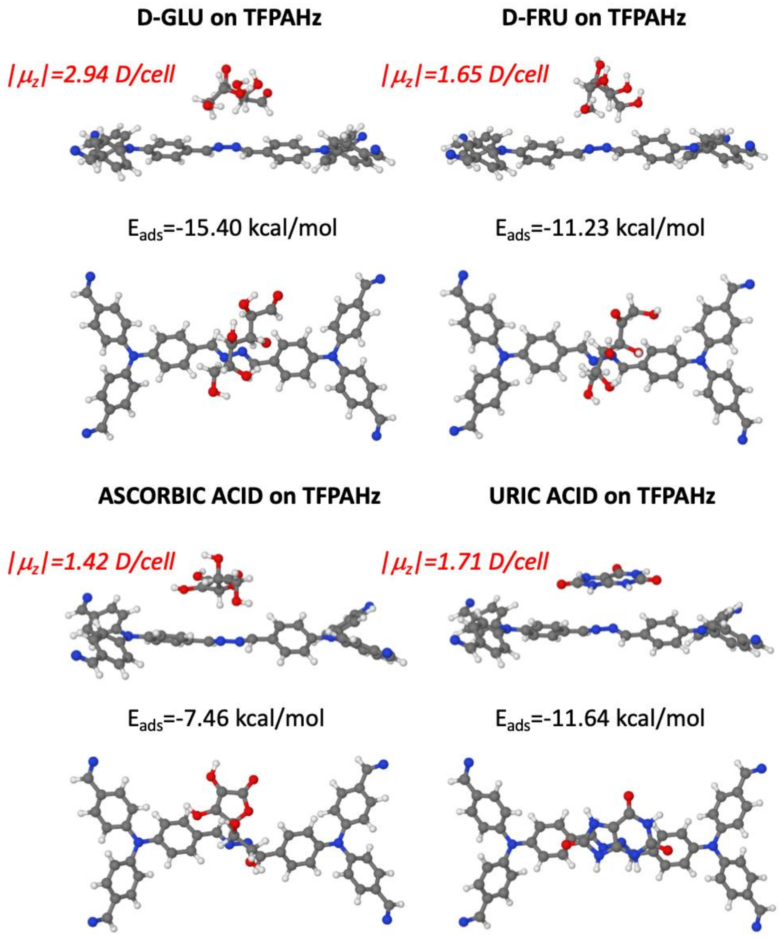

Figure S49. DFT-optimized ground-state geometries of glucose, fructose, ascorbic acid, and uric acid adsorbed on the TFPAHz surface, each is shown from side (top row) and top (bottom row) views. The adsorption energies (E_{ads}) and induced out-of-plane dipole moments ($|\mu_z|$) are provided for each analyte. Glucose shows the strongest adsorption (-15.40 kcal/mol) and the highest dipolar perturbation (2.94 D/cell), whereas fructose (-11.23 kcal/mol, 1.65 D), ascorbic acid (-7.46 kcal/mol, 1.42 D), and uric acid (-11.64 kcal/mol, 1.71 D) exhibit significantly weaker adsorption and dipole induction. These results support the experimentally observed selectivity, wherein only glucose induces a sufficiently strong perturbation in the interfacial electrostatic environment to elicit a pronounced impedance response.

S-13: References

(1) Eldamak, A. R.; Thorson, S.; Fear, E. C. Study of the Dielectric Properties of Artificial Sweat Mixtures at Microwave Frequencies. *Biosensors* **2020**, *10* (6), 62.

(2) Boumya, W.; Laghrib, F.; Lahrich, S.; Farahi, A.; Achak, M.; Bakasse, M.; El Mhammedi, M. A. Electrochemical Impedance Spectroscopy Measurements for Determination of Derivatized Aldehydes in Several Matrices. *Heliyon* **2017**, *3* (10), e00392.

(3) Cui, F.; Sun, H.; Yang, X.; Zhou, H.; Wu, Y.; Li, J.; Li, H.; Liu, J.; Zeng, C.; Qu, B.; Zhang, J.; Zhou, Q. Laser-Induced Graphene (LIG)-Based Au@CuO/V2CTx MXene Non-Enzymatic Electrochemical Sensors for the Urine Glucose Test. *Chem. Eng. J.* **2023**, *457*, 141303.

(4) Yang, Y.; Shen, Y.; Wang, L.; Song, Y.; Wang, L. Three-Dimensional Porous Carbon/Covalent-Organic Framework Films Integrated Electrode for Electrochemical Sensors. *J. Electroanal. Chem.* **2019**, *855*, 113590.

(5) Zhou, H.; Zheng, M.; Tang, H.; Xu, B.; Tang, Y.; Pang, H. Amorphous Intermediate Derivative from ZIF-67 and Its Outstanding Electrocatalytic Activity. *Small* **2020**, *16* (2), 1904252.

(6) Zhang, Y.; Xu, J.; Xia, J.; Zhang, F.; Wang, Z. MOF-Derived Porous Ni_2P/Graphene Composites with Enhanced Electrochemical Properties for Sensitive Nonenzymatic Glucose Sensing. *ACS Appl. Mater. Interfaces* **2018**, *10* (45), 39151–39160.

(7) Liang, H.; Luo, Y.; Xiao, Y.; Chen, R.; Wang, L.; Song, Y. Ni/NiO/Carbon Derived from Covalent Organic Frameworks for Enzymatic-Free Electrochemical Glucose Sensor. *Ceram. Int.* **2024**, *50* (1), 977–984.

(8) Zhang, L.; Ding, Y.; Li, R.; Ye, C.; Zhao, G.; Wang, Y. Ni-Based Metal–Organic Framework Derived Ni@C Nanosheets on a Ni Foam Substrate as a Supersensitive Non-Enzymatic Glucose Sensor. *J. Mater. Chem. B* **2017**, *5* (28), 5549–5555.

(9) Zhou, Y.; Hu, Q.; Yu, F.; Ran, G.-Y.; Wang, H.-Y.; Shepherd, N. D.; D'Alessandro, D. M.; Kurmoo, M.; Zuo, J.-L. A Metal–Organic Framework Based on a Nickel Bis(Dithiolene) Connector: Synthesis, Crystal Structure, and Application as an Electrochemical Glucose Sensor. *J. Am. Chem. Soc.* **2020**, *142* (48), 20313–20317.

(10) Zhang, Q.; Li, P.; Wu, J.; Peng, Y.; Pang, H. Pyridine-Regulated Lamellar Nickel-Based Metal–Organic Framework (Ni-MOF) for Nonenzymatic Electrochemical Glucose Sensor. *Adv. Sci.* **2023**, *10* (27), 2304102.

(11) Shi, X.; Ling, Y.; Li, Y.; Li, G.; Li, J.; Wang, L.; Min, F.; Hübner, R.; Yuan, S.; Zhan, J.; Cai, B. Complete Glucose Electrooxidation Enabled by Coordinatively Unsaturated Copper Sites in Metal–Organic Frameworks. *Angew. Chem. Int. Ed.* **2023**, *62* (51), e202316257.

(12) Zhang, J.; Chen, L.; Yang, K. In Situ Synthesis of CuO Nanoparticles Decorated Hierarchical Ce-Metal-Organic Framework Nanocomposite for an Ultrasensitive Non-Enzymatic Glucose Sensor. *Ionics* **2019**, *25* (9), 4447–4457.

(13) Li, X.; Niu, X. H.; Wu, H. Y.; Meng, S. C.; Zhang, W. C.; Pan, J. M.; Qiu, F. X. Impedimetric Enzyme-Free Detection of Glucose via a Computation-Designed Molecularly Imprinted Electrochemical Sensor Fabricated on Porous Ni Foam. *Electroanalysis* **2017**, *29* (5), 1243–1251.

(14) Deo, M.; Sahoo, D.; Kar, P. Self-Assembled Monolayer of Poly(*o* - Phenylenediamine)/Silver Core–Shell Hybrid-Based Enzyme-Free Impedimetric Glucose Sensor for Blood Samples. *RSC Adv.* **2024**, *14* (37), 26863–26872.

(15) Wu, H.; Yan, Y.; Huang, Q.; Liang, G.; Qiu, F.; Ye, Z.; Liu, D. A Simple, Cost-Effective and Selective Analysis of Glucose *via* Electrochemical Impedance Sensing Based on Copper and Nitrogen Co-Doped Carbon Quantum Dots. *New J. Chem.* **2020**, *44* (29), 12723–12728.

(16) Kannathvalappil, A.; A. S., V.; Bhat, N.; Gupta, N. K.; Pandey, K. Impedance Spectroscopy Unveils Interfacial Dynamics in NiO-Modified Electrodes for Glucose Detection. *J. Phys. Chem. C* **2025**, *129* (15), 7387–7401.

(17) McLean, A. D.; Chandler, G. S. Contracted Gaussian Basis Sets for Molecular Calculations. I. Second Row Atoms, Z=11–18. *J. Chem. Phys.* **1980**, *72* (10), 5639–5648.

(18) Krishnan, R.; Binkley, J. S.; Seeger, R.; Pople, J. A. Self-Consistent Molecular Orbital Methods. XX. A Basis Set for Correlated Wave Functions. *J. Chem. Phys.* **1980**, *72* (1), 650–654.

(19) Zhao, Y.; Truhlar, D. G. The M06 Suite of Density Functionals for Main Group Thermochemistry, Thermochemical Kinetics, Noncovalent Interactions, Excited States, and Transition Elements: Two New Functionals and Systematic Testing of Four M06-Class Functionals and 12 Other Functionals. *Theor. Chem. Acc.* **2008**, *120* (1–3), 215–241.

(20) Giannozzi, P.; Baroni, S.; Bonini, N.; Calandra, M.; Car, R.; Cavazzoni, C.; Ceresoli, D.; Chiarotti, G. L.; Cococcioni, M.; Dabo, I.; Dal Corso, A.; De Gironcoli, S.; Fabris, S.; Fratesi, G.; Gebauer, R.; Gerstmann, U.; Gougoussis, C.; Kokalj, A.; Lazzeri, M.; Martin-

Samos, L.; Marzari, N.; Mauri, F.; Mazzarello, R.; Paolini, S.; Pasquarello, A.; Paulatto, L.; Sbraccia, C.; Scandolo, S.; Sclauzero, G.; Seitsonen, A. P.; Smogunov, A.; Umari, P.; Wentzcovitch, R. M. QUANTUM ESPRESSO: A Modular and Open-Source Software Project for Quantum Simulations of Materials. *J. Phys. Condens. Matter* **2009**, *21* (39), 395502.

(21) Perdew, J. P.; Ruzsinszky, A.; Csonka, G. I.; Vydrov, O. A.; Scuseria, G. E.; Constantin, L. A.; Zhou, X.; Burke, K. Restoring the Density-Gradient Expansion for Exchange in Solids and Surfaces. *Phys. Rev. Lett.* **2008**, *100* (13), 136406.

(22) Grimme, S. Semiempirical GGA-type Density Functional Constructed with a Long-range Dispersion Correction. *J. Comput. Chem.* **2006**, *27* (15), 1787–1799.

(23) Rappe, A. M.; Rabe, K. M.; Kaxiras, E.; Joannopoulos, J. D. Optimized Pseudopotentials. *Phys. Rev. B* **1990**, *41* (2), 1227–1230.

(24) Pack, J. D.; Monkhorst, H. J. "Special Points for Brillouin-Zone Integrations"—a Reply. *Phys. Rev. B* **1977**, *16* (4), 1748–1749.

(25) *The Hubbard Model: A Reprint Volume*; Montorsi, A., Ed.; World Scientific: Singapore ; New Jersey, 1992.

(26) Tada, K.; Kitagawa, Y. Issues on DFT+ *U* Calculations of Organic Diradicals. *Phys. Chem. Chem. Phys.* **2023**, *25* (46), 32110–32122.

(27) Henkelman, G.; Uberuaga, B. P.; Jónsson, H. A Climbing Image Nudged Elastic Band Method for Finding Saddle Points and Minimum Energy Paths. *J. Chem. Phys.* **2000**, *113* (22), 9901–9904.
